# A Cardiac‐Targeting and Anchoring Bimetallic Cluster Nanozyme Alleviates Chemotherapy‐Induced Cardiac Ferroptosis and PANoptosis

**DOI:** 10.1002/advs.202405597

**Published:** 2024-10-28

**Authors:** Junyue Xing, Xiaohan Ma, Yanan Yu, Yangfan Xiao, Lu Chen, Weining Yuan, Yingying Wang, Keyu Liu, Zhiping Guo, Hao Tang, Kelong Fan, Wei Jiang

**Affiliations:** ^1^ National Health Commission Key Laboratory of Cardiovascular Regenerative Medicine Central China Subcenter of National Center for Cardiovascular Diseases Henan Cardiovascular Disease Center Fuwai Central‐China Cardiovascular Hospital Central China Fuwai Hospital of Zhengzhou University Zhengzhou 450046 China; ^2^ Henan Key Laboratory of Chronic Disease Management Central China Fuwai Hospital of Zhengzhou University Zhengzhou Henan 451464 China; ^3^ Zhengzhou Key Laboratory of Cardiovascular Aging Central China Fuwai Hospital of Zhengzhou University Zhengzhou Henan 451464 China; ^4^ Department of Cardiovascular Diseases the First Clinical Medical College Shanxi Medical University Taiyuan Shanxi 030001 China; ^5^ School of Clinical Medicine Shandong Second Medical University Weifang Shandong 261053 China; ^6^ CAS Engineering Laboratory for Nanozyme Key Laboratory of Biomacromolecules (CAS) CAS Center for Excellence in Biomacromolecules Institute of Biophysics Chinese Academy of Sciences Beijing 100101 China; ^7^ Nanozyme Laboratory in Zhongyuan Henan Academy of Innovations in Medical Science Zhengzhou Henan 451163 China; ^8^ Academy of Medical Sciences Tianjian Laboratory of Advanced Biomedical Sciences Zhengzhou University Zhengzhou Henan China

**Keywords:** antioxidant activity, bimetallic cluster nanozyme, DOX‐induced cardiotoxicity, ferroptosis, PANoptosis

## Abstract

Doxorubicin (DOX), a potent antineoplastic agent, is commonly associated with cardiotoxicity, necessitating the development of strategies to reduce its adverse effects on cardiac function. Previous research has demonstrated a strong correlation between DOX‐induced cardiotoxicity and the activation of oxidative stress pathways. This work introduces a novel antioxidant therapeutic approach, utilizing libraries of tannic acid and N‐acetyl‐L‐cysteine‐protected bimetallic cluster nanozymes. Through extensive screening for antioxidative enzyme‐like activity, an optimal bimetallic nanozyme (AuRu) is identified that possess remarkable antioxidant characteristics, mimicking catalase‐like enzymes. Theoretical calculations reveal the surface interactions of the prepared nanozymes that simulate the hydrogen peroxide decomposition process, showing that these bimetallic nanozymes readily undergo OH⁻ adsorption and O₂ desorption. To enhance cardiac targeting, the atrial natriuretic peptide is conjugated to the AuRu nanozyme. These cardiac‐targeted bimetallic cluster nanozymes, with their anchoring capability, effectively reduce DOX‐induced cardiomyocyte ferroptosis and PANoptosis without compromising tumor treatment efficacy. Thus, the therapeutic approach demonstrates significant reductions in chemotherapy‐induced cardiac cell death and improvements in cardiac function, accompanied by exceptional in vivo biocompatibility and stability. This study presents a promising avenue for preventing chemotherapy‐induced cardiotoxicity, offering potential clinical benefits for cancer patients.

## Graphical abstract

This study introduces an innovative antioxidant bimetallic cluster nanozyme designed with cardiac‐targeting and anchoring capabilities, effectively mitigating chemotherapy‐induced cardiac ferroptosis and PANoptosis.

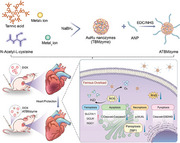

## 1 Introduction

Doxorubicin (DOX), an anthracycline drug, is extensively utilized in clinical tumor chemotherapy.^[^ ^1^ ^]^ However, its use is associated with the risk of cardiotoxicity, resulting in the onset of progressive, chronic, and potentially life‐threatening cardiomyopathy, referred to as DOX‐induced cardiomyopathy (DIC).^[^ ^2^, ^3^ ^]^ DIC is a severe form of cardiomyopathy with a significantly worse prognosis compared to other types of cardiomyopathy. The cardiotoxicity associated with DIC limits the clinical use of doxorubicin in treating malignant tumors.^[^ ^4^ ^]^ Several studies have reported potential molecular mechanisms of DIC, including transcriptional dysregulation mediated by topoisomerase IIβ (Top2b) inhibition, disruptions in calcium ion handling, mitochondrial iron accumulation, mitochondrial dysfunction, and alteration of cell death pathways.^[^ ^5^, ^6^ ^]^ DOX accumulates in the nuclei and mitochondria of cardiomyocytes, leading to mitochondrial iron overload and excessive oxidative stress. This, in turn, disrupts intracellular death signaling pathways, including ferroptosis^[^ ^7^, ^8^, ^9^ ^]^ and PANoptosis.^[^ ^10^, ^11^, ^12^, ^13^, ^14^ ^]^ These related molecular processes have been identified as potential pathogenesis of DIC. The exact mechanism of cardiac injury induced by DOX remains unclear, and there are currently no established safe and effective treatments to prevent this damage. Therefore, finding an effective way to reduce or prevent the cardiac side effects of chemotherapy is essential.

Previous research has demonstrated that natural enzymes such as manganese superoxide dismutase (SOD) and glutathione peroxidase1 (GPx1) have a significant therapeutic effect on DIC,^[^ ^15^, ^16^ ^]^ reducing the level of reactive oxygen species (ROS) in cells and decrease ferroptosis, thereby alleviating cardiac chemotherapy toxicity.^[^ ^17^ ^]^ However, the clinical potential of natural antioxidant enzymes is limited by several drawbacks, including rapid inactivation, degradation, short half‐life, and poor membrane permeability^[^ ^18^ ^]^ Nanozymes^[^ ^19^, ^20^, ^21^ ^]^ a novel type of nanomaterials with biocatalytic functions, exhibit high catalytic activity and biocompatibility. They have been demonstrated to repair and protect heart function through multiple mechanisms, including immune response modulation, free radical scavenging, and the reduction of oxidative stress.^[^ ^22^, ^23^, ^24^, ^25^ ^]^ Investigating the role of nanozymes in regulating programmed cell death in myocardial cells caused by chemotherapy, and their potential to mitigate cardiac toxicity, is crucial for improving the safety and efficacy of chemotherapy. Gold (Au) exhibits exceptional biocompatibility and adjustable enzyme‐like activity, making it widely utilized for the eradication of tumors^[^ ^26^, ^27^ ^]^ and inflammation.^[^ ^28^ ^]^ One significant challenge in using nanozymes for treating cardiac chemotherapy toxicity is the rapid blood flow in cardiac tissue, which reduces drug residence time and diminishes the effectiveness of current antioxidant therapies. Therefore, developing an effective cardiac‐targeted nanozyme with anchoring capability is essential for treating patients with chemotherapy‐induced cardiac toxicity.

**Scheme 1:**
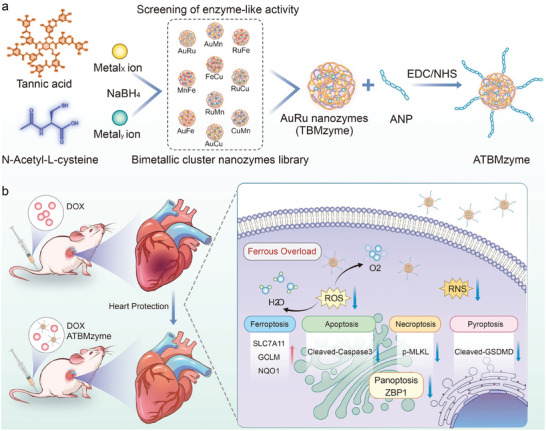
Schematic illustration of a) preparation of bimetallic cluster nanozyme library and cardiac‐targeted ATBMzyme and b) application for the treatment of DIC by the cardiac‐targeted ATBMzyme.

This study aims to investigate how nanozymes regulate cardiac ferroptosis and PANoptosis and evaluate their potential to mitigate chemotherapy‐induced cardiomyopathy. A library of tannic acid (TA) and N‐acetyl‐L‐cysteine (NAC)‐protected bimetallic cluster nanozymes was synthesized. Through screening for antioxidative enzyme‐like activity, the optimal NAC‐protected bimetallic nanozyme (AuRu) was identified. The synthesis, characterization, and biocatalytic properties of the synthesized nanozymes were explored, and animal experiments were conducted. Cardiac‐tissue‐targeting polyphenol (TA)^[^ ^29^ ^]^ engineering was employed to precisely construct a NAC‐protected bimetallic cluster nanozyme (TBMzyme) with ultrahigh antioxidative catalytic activity and heart‐targeting capabilities (**Scheme** **1**). To further enhance heart targeting and anchoring therapy, atrial natriuretic peptide (ANP) was conjugated to TBMzyme, creating ATBMzyme. These findings provide scientific evidence for the potential clinical application of nanozymes in reducing chemotherapy‐induced cardiac toxicity and suggest a targeted approach to alleviating cardiac ferroptosis and PANoptosis, potentially revolutionizing the treatment of DIC.

## 2 Results and Discussion

### 2.1 Synthesis, Characterization, and Screening of ATBMzyme

**Figure 1:**
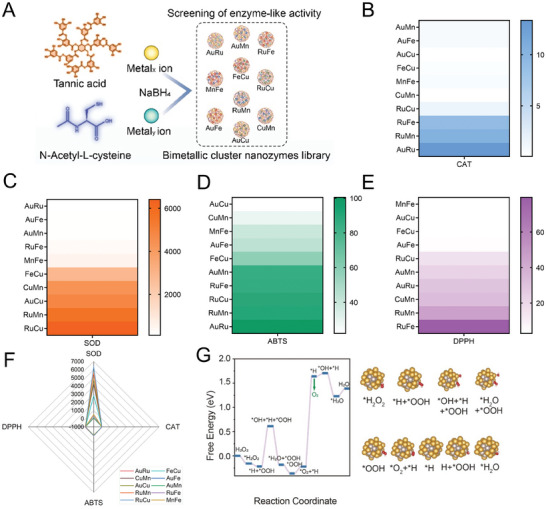
Preparation and screen of bimetallic nanozymes. A) Schematic representation of the preparation of different bimetallic nanozymes. B) The CAT‐like activity of different bimetallic nanozymes. C) The SOD‐like activity of different bimetallic nanozymes. D) ABTS radical scavenging ratio of different bimetallic nanozymes. E) DPPH radical scavenging ratio of different bimetallic nanozymes. F) Radar chart (SOD/CAT‐like activity, ABTS, and DPPH radical scavenging of different bimetallic nanozymes. G) Surface configurations of different modeled initial, transition, and final states for the simulated CAT‐like catalytic processes.

Initially, various bimetallic nanozymes (AuRu, AuFe, AuMn, RuFe, MnFe, FeCu, CuMn, AuCu, RuMn, and RuCu) were synthesized using ligand interaction approaches, with NAC, TA, metal_x_ ion, and metal_y_ ion serving as ligands (**Figure** **1**A). The SOD/catalase (CAT)‐like activities, as well as the 2,2′‐azino‐bis(3‐ethylbenzothiazoline‐6‐sulfonic acid) diammonium salt (ABTS) and 1,1‐diphenyl‐2‐picrylhydrazyl (DPPH) radical scavenging ratios of various bimetallic nanozymes, have been depicted in Figure 1B–F. The data suggests that the AuRu bimetallic nanozymes (TBMzyme) exhibit improved CAT‐like enzyme activity, nitrogen‐free radical scavenging ability, and potential biosafety compared to other bimetallic nanozymes. Given the outstanding CAT‐like enzyme activity of TBMzyme (AuRu), theoretical calculations were performed to elucidate the mechanism by which the nanozyme mimics hydrogen peroxide decomposition on its surface. The step diagram for H₂O₂ decomposition on the TBMzyme is shown in Figure 1G, highlighting two main processes: the initial OH⁻ adsorption and O₂ desorption. The energy barrier for O₂ desorption is significantly high, indicating that O₂ produced during decomposition on the pure alloy surface tends to oxidize the alloy. This suggests a strong reducibility of the alloy, making initial O₂ production challenging. In the actual reaction, small alloy particles are oxidized by H₂O₂; only after reaching a certain level of oxidation does their reducibility decrease, making O₂ production easier. The energy barrier for the initial OH⁻ adsorption is relatively low (and even lower for the second OH⁻ adsorption). Electron and proton‐coupled reactions may also directly generate H₂O through the Eley‐Rideal mechanism. Thus, in this system, aside from the oxygen production step, all other steps occur readily. These findings suggest that TBMzyme (AuRu) has the potential to eliminate both ROS and reactive nitrogen species (RNS), making it a promising antioxidant agent for treating DIC.

**Figure 2:**
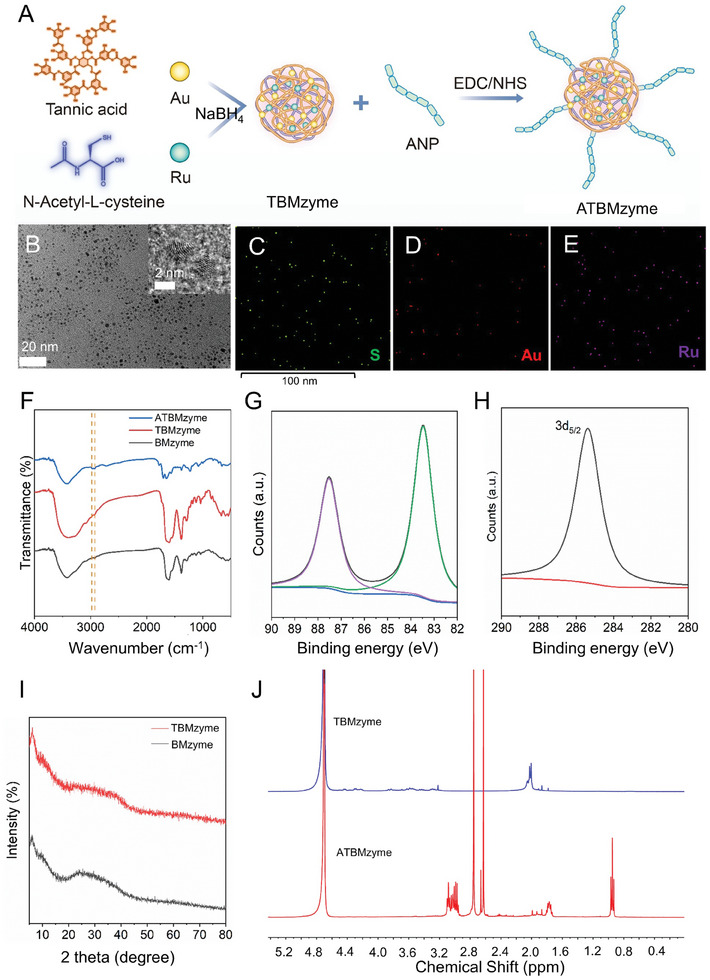
Preparation and characterization of nanozymes. A) Scheme representing the preparation of ATBMzyme. B) TEM image of ATBMzyme (Scale bar = 20 nm, inset scale bar of TEM is 2 nm). C–E) Elemental mapping of ATBMzyme. F) FTIR of BMzyme, TBMzyme, and ATBMzyme. G) XPS spectra of Au element in ATBMzyme. H) XPS spectra of Ru 3*d* in ATBMzyme. I) PXRD of BMzyme and TBMzyme. J) ^1^H NMR of TBMzyme, and ATBMzyme.

**Figure 3:**
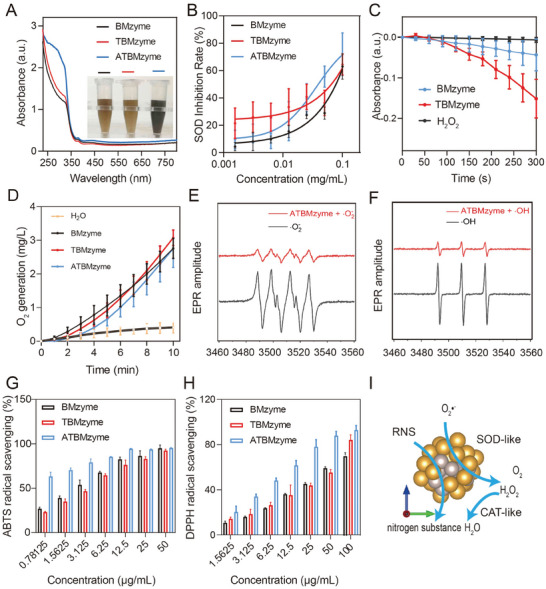
Analysis of the ROS scavenging potential of nanozyme. A) UV–vis spectrum of BMzyme, TBMzyme, and ATBMzyme in buffer solution. B) SOD‐like activities of BMzyme, TBMzyme, and ATBMzyme after incubation for different concentration in buffer solution (pH 7.4). C) The decomposition of H_2_O_2_ by BMzyme and TBMzyme was assessed after incubation in a buffer solution at pH 7.4 by monitoring absorbance changes at 240 nm. D) CAT‐like activities of BMzyme, TBMzyme, and ATBMzyme after incubation in buffer solution (pH 7.4) for various durations. E,F) ATBMzyme reduces the generation of superoxide radical (·O_2_^−^) and hydroxyl radical (·OH), illustrated by ESR spectroscopy. G) ABTS•+ scavenging rate of BMzyme, TBMzyme, ATBMzyme. H) DPPH• scavenging rate of BMzyme, TBMzyme, ATBMzyme. I) Schematic representation of cascade ROS and RNS scavenging activities of ATBMzyme. n = 3, data represent means ± SD.

Based on these results, TBMzyme was selected for modification with heart‐targeting ANP in the subsequent experiments (**Figure** **2**A). Transmission electron microscopy (TEM) images demonstrated that ATBMzyme exhibited a uniformly spherical nanoparticle morphology, with an average particle size of 3–5 nm (Figure 2B). Elemental mapping of ATBMzyme confirmed the presence of Au, Ru, and S in the composite, validating the successful synthesis of ATBMzyme (Figure 2C–E). The ratio of Au to Ru elements in ATBMzyme, as determined by inductively coupled plasma mass spectrometry (ICP‐MS), was 6:1 (Figure S1, Supporting Information). Dynamic Light Scattering (DLS) analysis revealed that the mean zeta potential of TBMzyme increased from ≈−42.28 to −3.72 mV after the addition of ANP, indicating the successful modification of TBMzyme to ATBMzyme (Figure S2, Supporting Information). The emergence of new absorption peaks detected by Fourier Transform Infrared Spectroscopy (FT‐IR) indicates the successful bonding of ANP to the surface, as depicted in Figure 2F. The surface chemistry of TBMzyme was analyzed through X‐ray photoelectron spectroscopy (XPS), showing absorption peaks of Au and Ru elements, as demonstrated in Figure 2G,H. The high‐resolution XPS spectra of Au orbitals revealed peaks at a binding energy of 83.7 eV, corresponding to zero‐valent Au. The XPS spectra of Ru orbitals displayed peaks for Ru 3*d*_5/2_, 3*p*_1/2,_ and 3*p*_3/2_ at a binding energy of 285.4, 485.5, and 462.9 eV, confirming the presence of oxidized Ru on ATBMzyme (Figure 2H; Figure S3, Supporting Information). Powder X‐ray diffraction (PXRD) analysis further showed that both TBMzyme and ATBMzyme exist in amorphous form (Figure 2I). The successful coupling of ANP to TBMzyme was confirmed by nuclear magnetic resonance (^1^H NMR, Figure 2J). Moreover, the UV–vis absorption spectrum of ATBMzyme was distinctly different from that of TBMzyme and BMzyme, indicating successful ANP conjugation (**Figure** **3**A). These results demonstrate the successful synthesis of a bimetallic cluster nanozyme conjugated with ANP.

After the successful synthesis of the ANP‐coupled bimetallic cluster nanozyme was confirmed, its antioxidant activity was investigated. Under neutral conditions, various enzyme‐like activities were exhibited by BMzyme, TBMzyme, and ATBMzyme. Figure 3B and Figure S4 (Supporting Information) show that SOD‐like activities were observed in these nanozymes when tested in a phosphate buffer at pH 7.4. Importantly, ATBMzyme exhibited the highest SOD‐like activity, quantitatively determined to be ≈913 U mg^−1^ through theoretical calculations. In contrast, BMzyme (0.01644 U/mg) and TBMzyme (0.065 U/mg) showed weak SOD‐like activity, suggesting that the inclusion of ANP protein components may provide additional active sites and enhance antioxidant capacity without affecting the hydrogen peroxide elimination ability of nanozyme, as illustrated in Figure 3C,D. Furthermore, as revealed by electron spin resonance (ESR) spectroscopy, ATBMzyme efficiently scavenged ·O_2_^−^ generated by the xanthine‐xanthine oxidase system (Figure 3E), as well as the ·OH produced by the Fenton reaction (Figure 3F). Consequently, ATBMzyme is capable of scavenging ·O_2_^−^, degrading H_2_O_2_, and promoting O_2_ production, indicating its ability to exert SOD/CAT cascade antioxidative activity.

The ability of BMzyme, TBMzyme, and ATBMzyme to remove nitrogen radicals was assessed using DPPH and ABTS free radical scavenging assays. Figure 3G and Figure S5B, D (Supporting Information) demonstrate that significant suppression of ABTS free radical generation was achieved by all three nanozymes. Their antioxidative effects suggest potential applications in treating diseases related to nitrogen radicals. Similarly, the DPPH assay demonstrated that ATBMzyme exhibited the strongest scavenging effect on hydroxyl radicals (Figure 3H; Figure S5A, C, Supporting Information). These findings highlight the importance of ANP and TA coordination in regulating the enzymatic activities of the nanozymes (Figure 3I). Lower hemolysis was observed for the carrier ATBMzyme at a concentration of 500 µg mL^−1^ (Figure S6, Supporting Information), suggesting that the carrier did not adversely affect erythrocyte integrity. This study suggests that the ATBMzyme exhibited potent antioxidant properties and minimal hemolytic activity, indicating its potential for alleviating DOX‐induced cardiomyocyte injury.

### 2.2 ATBMzyme Significantly Alleviates DOX‐Induced Ferroptosis and PANopotosis In Vitro

**Figure 4:**
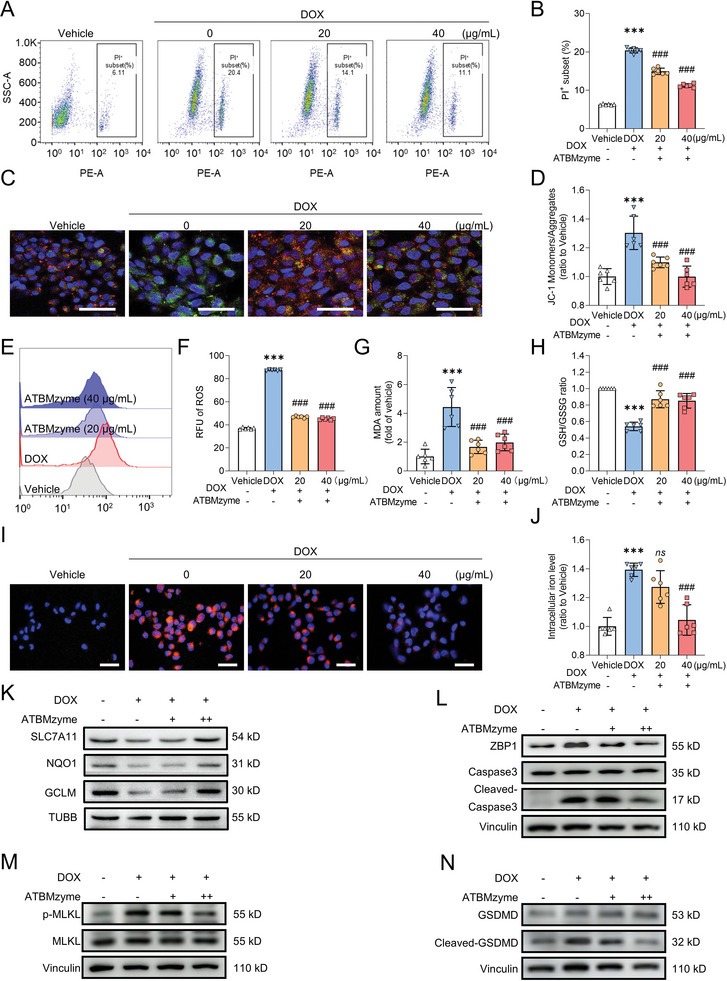
ATBMzyme significantly alleviates Doxorubicin‐induced ferroptosis and PANoptosis in vitro. AC16 cells were pre‐treated with ATBMzyme for 20 h, then exposed to DOX (800 nm) for 24 h. The cells were subsequently collected for the following experiments A) Flow cytometric calculation of the percentage of PI^+^ AC16 cells (n = 6). B) Quantification of PI staining of AC16 (n = 6). C) Representative micrographs of mitochondrial membrane potential (JC‐1 staining) in AC16, Green: JC‐1 monomer; Red: JC‐1 aggregates (scale bars, 50 µm; n = 6). D) Quantification of JC‐1 staining in AC16 (n = 6). E) Representative flow cytometry graphs of intracellular ROS (DCFH‐DA staining) in AC16 (n = 6). F) Quantification of intracellular ROS (DCFH‐DA staining) in AC16 (n = 6). G) Relative MDA levels in AC16 (n = 6). H) GSH/GSSH ratio in AC16 (n = 6). I) Representative micrographs of intracellular iron level (FerroOrange staining) in AC16 (scale bars, 50 µm; n = 6). J) Quantification of FerroOrange staining of AC16 (n = 6). K) Representative immunoblots of ferroptosis‐related proteins in AC16 (n = 6). L–N) Representative immunoblots of PANoptosis‐related proteins in AC16 (n = 6). Data has been expressed as mean ± SD and analyzed using one‐way ANOVA followed by Tukey's post hoc test, ^***^ *p* < 0.001 versus the Vehicle group; ^###^ *p* < 0.001 versus the DOX group.

The intracellular antioxidant efficacy of nanozymes depends on their ability to evade lysosomal degradation. To evaluate this capability, we quantified the lysosomal escape proficiency of ATBMzyme using confocal laser scanning microscopy (CLSM). The results showed that Cy5.5‐tagged ATBMzyme began to colocalize with lysosomal compartments within 1 hour of incubation, achieving the highest degree of colocalization at 2 hours. After a 4‐hour incubation, ATBMzyme exhibited the ability to escape lysosomal confinement, remaining in the cytosol until it was extruded from the cell approximately 8 hours later. These observations confirm the strong lysosomal escape capacity of our bimetallic nanozyme, which is crucial for its antioxidant function in the cellular environment (Figure S7A, Supporting Information). Next, we examined how ATBMzyme protects against DOX‐induced myocardial injury in human cardiomyocyte AC16 cells. Flow cytometry analysis showed that pretreatment with ATBMzyme significantly reduced DOX‐induced cell death in a dose‐dependent manner (**Figure** **4**A,B).

DOX accumulates in cardiac mitochondria, causing oxidative stress and mitochondrial dysfunction, which are major contributors to DOX‐induced cardiotoxicity.^[^ ^29^ ^]^ Mitochondrial function was further evaluated using JC‐1 staining. The results revealed that DOX‐induced AC16 cells exhibited a reduced mitochondrial membrane potential (evidenced by JC‐1 staining turning green), while pretreatment with ATBMzyme restored a portion of the membrane potential (Figure 4C,D). Furthermore, treatment with ATBMzyme reduced DOX‐induced intracellular ROS generation (Figure 4E,F), further demonstrating its antioxidant capabilities.

Intracellular iron accumulation stimulates the production of reactive oxygen species (ROS) and activates lipoxygenases, which cause damage to cell and mitochondrial membranes, ultimately resulting in ferroptosis. Mitochondria‐specific antioxidants have shown efficacy in reducing damage‐induced cardiomyopathy (DIC), underscoring the pivotal role of oxidative stress on mitochondria as a key mechanism in ferroptosis‐mediated cardiac damage.^[^ ^9^, ^10^, ^14^, ^30^ ^]^ Therefore, markers associated with ferroptosis were evaluated. It was demonstrated that pre‐treatment with ATBMzyme resulted in reduced levels of lipid peroxide malondialdehyde (MDA) (Figure 4G), an increased glutathione (GSH)/oxidized glutathione (GSSG) ratio (Figure 4H), lowered iron accumulation (Figure 4I,J), dereased of ferrous ions and lipid peroxide level in mitochondria (Figure S7B,C,Supporting Information), and elevated protein levels of SLC7A11, GCLM, and NQO1 (Figure 4K; Figure S7D, Supporting Information). These findings indicate that ATBMzyme has the potential to target ferroptosis and alleviate DOX‐induced cardiomyocyte injury.

PANoptosis, as a form of programmed cell death characterized by oxidative stress and mitochondrial dysfunction, encompasses three types of cell death: pyroptosis, apoptosis, and necroptosis, and has also been reported to be involoved in the occurrence of DIC.^[^ ^36^ ^]^ In our study, reduced expression of PANoptosis core response protein Z‐DNA‐binding protein 1 (ZBP1), apoptosis marker cleaved caspase‐3, necroptosis marker p‐MLKL, and pyroptosis marker cleaved GSDMD was observed in the groups pre‐treated with ATBMzyme compared to the DOX group (Figure 4L–N; Figure S7E, Supporting Information). This suggests that DOX‐induced PANoptosis can be inhibited by ATBMzyme. These results indicate that ATBMzyme alleviates mitochondrial damage and reduces both ferroptosis and PANoptosis in the context of DOX‐induced cardiac injury.

### 2.3 ATBMzyme Alleviates DOX‐Induced Cardiotoxicity by Inhibiting Ferroptosis and PANoptosis

**Figure 5:**
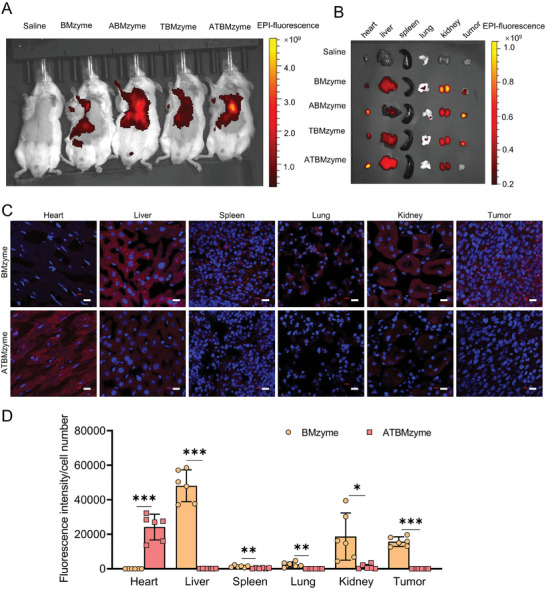
Heart‐specific targeting ability of BMzyme and ATBMzyme. A) Representative image of live imaging of animals. B) Representative fluorescent image of heart, liver, spleen, lung, kidney and tumor. C) Representative immunofluorescence images, and D) fluorescence intensity quantification of the distribution of BMzyme and ATBMzyme in the tissues from different organs (scale bars, 50 µm; n = 6). Data were expressed as mean ± SD and analyzed using a two‐tailed Student's t‐test, ^*^ *p* < 0.05, ^**^ *p* < 0.01, ^***^ *p* < 0.001.

To confirm the inhibitory effect of ATBMzyme on DOX‐induced cardiac injury in vivo, the cardiac targeting efficacy of the nanozymes was first assessed using live imaging of animals. Cy5.5‐tagged BMzyme ABMzyme, TBMzyme, and ATBMzyme was administrated via the tail vein to 4T1‐bearing Balb/c mice. After 24 h, the image indicated ATBMzymes, and ABMzymes were significantly enriched in the heart (**Figure** **5**A,B). Immunofluorescence of paraffin sections showed significant enrichment of Cy5.5‐tagged ATBMzyme in the heart, while Cy5.5‐tagged BMzyme was evenly distributed across various organs. Off‐target fluorescence was rarely observed in the tumors of the ATBMzyme‐treated groups (Figure 5B–D). These results confirm the heart‐targeting effect of ATBMzyme. Pharmacokinetics were analyzed using ICP‐MS to measure Ru ion content in the blood (Figure S8, Supporting Information). The analysis showed that approximately half of the drugs were metabolized within 4 h, indicating potential safety for the drugs.

**Figure 6:**
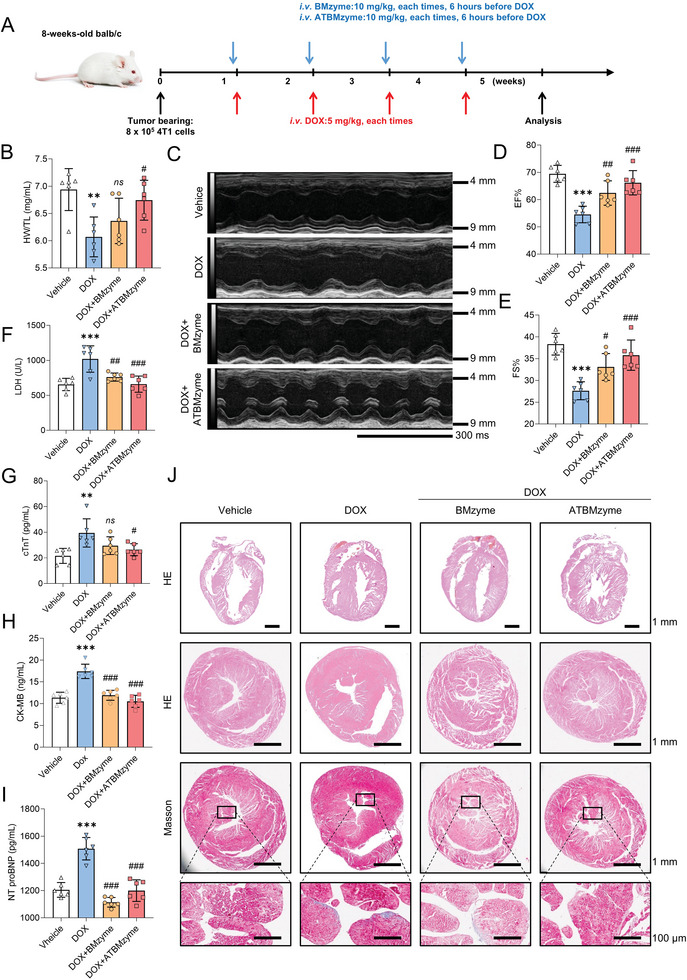
Cardiac‐targeting ATBMzyme significantly alleviates Doxorubicin‐induced myocardial injury and reduces cardiac function. A) Experiment design: A chronic DIC mouse model was established in Babl/c mice bearing 4T1 tumor cells, BMzyme and ATBMzyme were used for the treatment of the DIC model. All tests and analyses were implemented 1 week after the last DOX injection. B) Heart weight/Tibial length ratio (n = 6). C) Representative echocardiographic images of cardiac function (scale bar = 300 ms, n = 6). D,E) Quantitative analysis of ejection fraction and fraction shortening. (n = 6). F–I) Serum levels of lactate dehydrogenase (LDH), cardiac troponin T (cTnT), creatine kinase‐myocardial band (CK‐MB), and N‐terminal pro‐B type natriuretic peptide (NT‐proBNP) to assess cardiac injury (n = 6). J) HE and Masson staining of heart from Figure 6A, scale bar were indicated in the Figure. Data were expressed as mean ± SD and analyzed using one‐way ANOVA followed by Tukey's post hoc test, ^**^ *p* < 0.01 and ^***^ *p* < 0.001 versus Vehicle group; ^#^p < 0.05, ^##^ *p* < 0.01 and ^###^ *p* < 0.001 versus DOX group.

The Balb/c mice model with 4T1 tumor cells was used to replicate the conditions of chemotherapy‐induced cardiotoxicity in a preclinical setting. The schematic representation is provided in **Figure** **6**A. At the endpoint, several indices were collected to evaluate cardiac function. DOX treatment led to decreased body weight (Figure S9B, Supporting Information) and HW/TL ratio (Figure 6B), as well as worsened cardiac dysfunction and myocardial injury, as evidenced by reduced ejection fraction (EF%) and fractional shortening (FS%) (Figure 6C–E) and increased levels of serum myocardial injury markers, including LDH, cTnT, CK‐MB, and NT‐proBNP (Figure 6F–I). Masson's trichrome staining (Figure 6J) indicated that ATBMzyme attenuated cardiac fibrosis in DOX‐treated mice. Additionally, targeted delivery of ATBMzyme to the heart was found to mitigate cardiac injury. Both BMzyme and cardiac‐targeting ATBMzyme were found to reduce weight loss, impaired cardiac function, and cardiac injury (Figure 6B‐J). Tumor volumes were measured every three days post‐4T1 injection (Figure S9A,C–E, Supporting Information), and tumor weights were assessed at the endpoint (Figure S9F, Supporting Information). Compared to the vehicle group, both BMzyme and ATBMzyme treatments resulted in smaller tumor volumes and weights. However, BMzyme demonstrated a less potent anti‐tumor effect compared to the DOX group. In contrast, ATBMzyme, when combined with ANP and TA, manifested a significant anti‐tumor effect similar to that of the DOX group (Figure S9A–F, Supporting Information). The data indicate that cardiac‐targeting ATBMzyme effectively protects the heart from DOX‐induced damage while maintaining its anti‐cancer efficacy.

**Figure 7:**
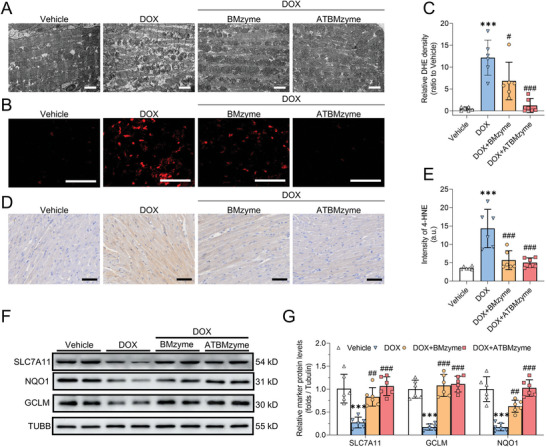
The cardiac‐targeting ATBMzyme significantly alleviates DIC by preventing ferroptosis‐induced myocardial cell death. A) Representative micrographs of heart tissues examined by transmission electron microscopy (scale bars, 2 µm). B) Representative micrographs of cardiac intracellular ROS levels (DHE staining) (scale bars, 50 µm; n = 6). C) Quantification of DHE staining in sections of heart tissues (n = 6). D) Representative immunohistochemistry micrographs of cardiac 4‐HNE staining (scale bars, 50 µm; n = 6). E) Quantification of 4‐HNE staining in heart sections (n = 6). F) Representative immunoblots of ferroptosis–related proteins in heart tissues (n = 6). G) Quantitative analysis of ferroptosis‐related proteins in heart tissues (n = 6). Data were expressed as mean ± SD and analyzed using one‐way ANOVA followed by Tukey's post hoc test, ^***^ *p* < 0.001 versus Vehicle group; ^#^ *p* < 0.05, ^##^ *p* < 0.01, ^###^ *p* < 0.001 versus DOX group.

**Figure 8:**
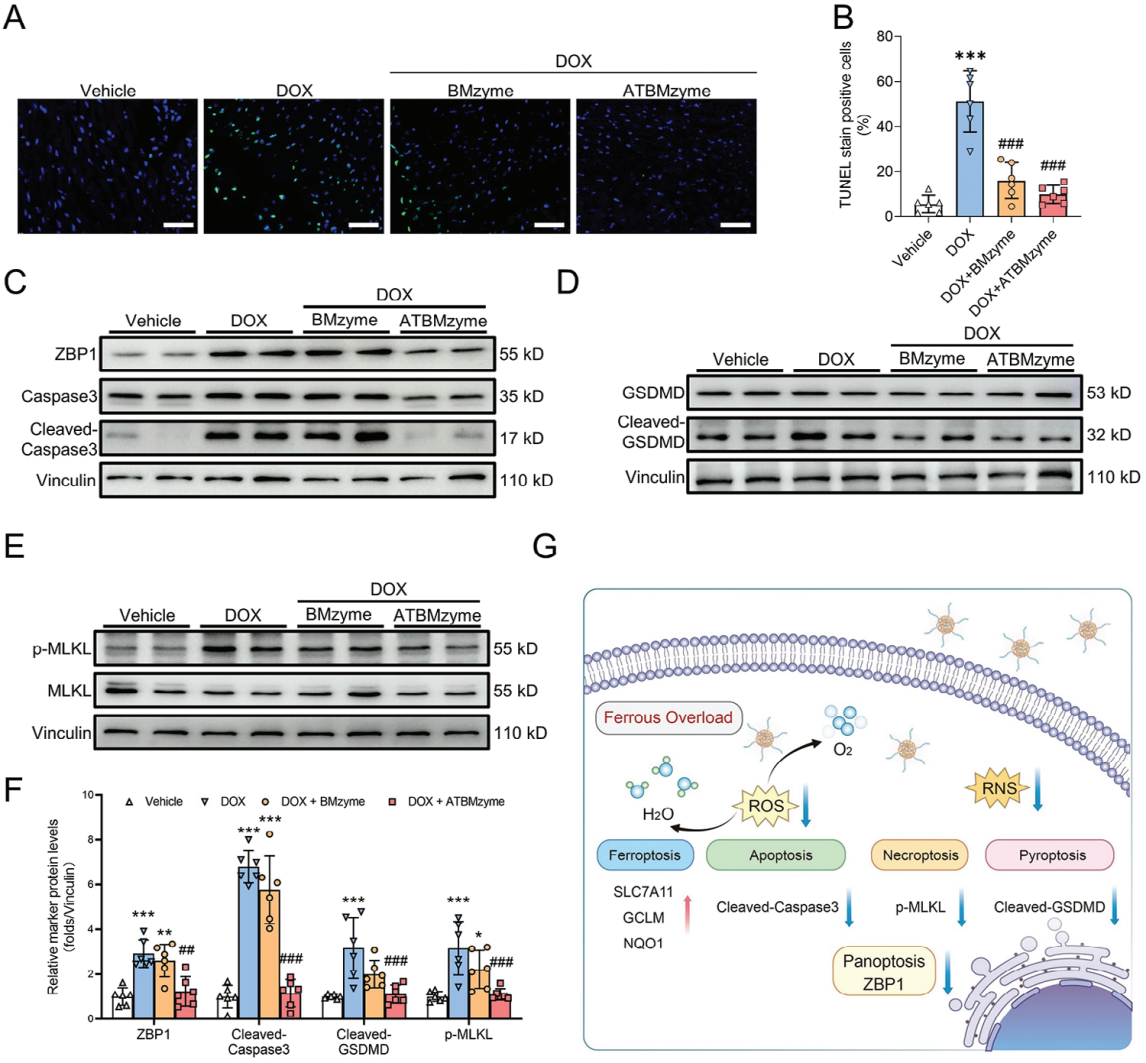
Cardiac‐targeting ATBMzyme significantly alleviates DIC, by preventing PANoptosis‐induced myocardial cell death. A) Representative images of TUNEL staining in heart sections, green fluorescence represents the TUNEL‐positive cells (scale bars, 50 µm; n = 6). B) Quantification of TUNEL staining in heart sections (n = 6). C–E) Representative immunoblots of PANoptosis‐related proteins in heart tissues (n = 6). F) Quantitative analysis of PANoptosis‐related proteins in heart tissues (n = 6). G) Schematic diagram depicting how ATBMzyme alleviates chemotherapy‐induced cardiac ferroptosis and PANoptosis. The data were expressed as mean ± SD and analyzed using one‐way ANOVA followed by Tukey's post hoc test, ^*^ *p*<0.05, ^**^ *p* < 0.01, ^***^ *p* < 0.001 versus Vehicle group; ^##^ *p* < 0.01, ^###^ *p* < 0.001 versus DOX group.

The mechanism of DOX‐induced changes in cardiomyocytes was investigated in vivo. Alterations in mitochondrial ultrastructure, including ridge breakage and vacuolization, were observed using transmission electron microscopy in the DOX group. Significant improvements in mitochondrial morphology were observed with both BMzyme and ATBMzyme treatments (**Figure** **7**A). Increased levels of intracellular ROS (Figure 7B,C). Total ROS (Figure S10, Supporting Information) and 4‐HNE (Figure 7D,E) were detected in DOX‐treated mice compared to the vehicle group. A significant decrease in the expression levels of anti‐ferroptosis‐related proteins SLC7A11, GCLM, and NQO1 was observed in the DOX group (Figure 7F,G). An increase in the number of apoptotic cardiomyocytes following DOX treatment was also observed (**Figure** **8**A,B), with significantly elevated levels of PANoptosis‐related proteins ZBP1, cleaved‐caspase 3, cleaved‐GSDMD, and p‐MLKL in the DOX group compared to the vehicle group (Figure 8C–F). Partial restoration of disrupted mitochondria, reduction in ROS generation, and effective suppression of ferroptosis and PANoptosis phenotypes were achieved with both BMzyme and ATBMzyme treatments. ATBMzyme demonstrated significant protective effects against DOX‐induced cardiomyocyte ferroptosis and PANoptosis, consistent with the in vitro results. Collectively, the data indicates that ATBMzyme treatment ameliorates doxorubicin‐induced cardiotoxicity by addressing ferroptosis and PANoptosis‐related molecular changes in the hearts of DIC mice (Figure 8G).

### 2.4 BMzyme and ATBMzyme Protect Against DOX‐Stimulated Hepatotoxicity and Nephrotoxicity

**Figure 9:**
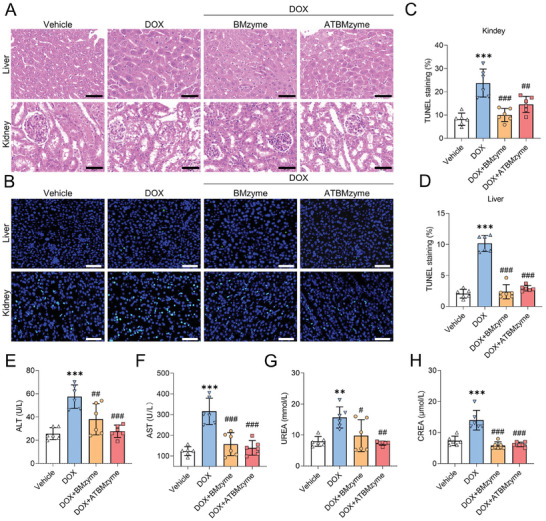
Both BMzyme and ATBMzyme show protection against DOX‐induced renal and liver damage. A) Representative micrographs of H&E staining in liver and kidney sections (scale bars, 50 µm; n = 6). B) Representative images of TUNEL staining in hepatic and renal sections, green fluorescence represents the TUNEL‐positive cells (scale bars, 50 µm; n = 6). Quantification of TUNEL staining in kidney C) and liver D). E–H) Serum levels of alanine aminotransferase (ALT), aspartate aminotransferase (AST), urea nitrogen (UREA), and creatinine (CREA) to assess hepatic and renal injury (n = 6). The data were expressed as mean ± SD and analyzed using one‐way ANOVA followed by Tukey's post hoc test, ^**^ *p* < 0.01 and ^***^ *p* < 0.001 versus Vehicle group; ^#^ *p* < 0.05, ^##^ *p* < 0.01, and ^###^ *p* < 0.001 versus DOX group.

Despite employing a targeted delivery approach, a minor amount of ATBMzyme still accumulates in normal tissues, particularly in the liver and kidneys, which are involved in drug metabolism. The distribution of BMzyme and ATBMzyme in these organs was visualized using immunofluorescence (Figure 5B–D). DOX‐induced hepatotoxicity and nephrotoxicity were confirmed through H&E staining of liver and kidney sections (**Figure** **9**A) and TUNEL staining, which revealed significant apoptosis in hepatocytes and renal cells (Figure 9B–D). Elevated levels of alanine aminotransferase (ALT) and aspartate aminotransferase (AST) were observed in the DOX group (Figure 9E,F), indicating hepatic damage, while increased levels of urea nitrogen (UREA) and creatinine (CREA) (Figure 9G,H) confirmed renal impairment. In contrast, liver and kidney tissues from the BMzyme and ATBMzyme groups exhibited fewer necrotic and apoptotic cells compared to those from the DOX group (Figure 9A–D). The serological parameters in the BMzyme and ATBMzyme groups were closer to baseline levels. Moreover, the H&E staining results of the heart, spleen, and lungs confirmed that compared to the Vehicle group, mice treated with DOX + ATBMzyme showed negligible side effects observed in the main organs (Figure S11, Supporting Information). In summary, the bimetallic (AuRu) cluster nanozyme demonstrated protective effects against DOX‐induced hepatotoxicity and nephrotoxicity.

Heart disease is a leading global cause of death, and the cardiac side effects induced by chemotherapy drugs have become a significant clinical concern.^[^ ^31^, ^32^ ^]^ Although chemotherapy is deemed crucial for cancer treatment, cardiac toxicity is the most common side effect. Doxorubicin‐induced cardiomyopathy (DIC) is identified as a severe form of cardiomyopathy with a poorer prognosis compared to other types, and the effectiveness of doxorubicin in treating malignant tumors is limited by its cardiotoxicity. Cardiac cells can be damaged by chemotherapy drugs through various mechanisms, including the generation of reactive oxygen species (ROS), disruption of calcium ion balance, and modulation of programmed cell death pathways. These effects may result in myocardial fibrosis, reduced cardiac function, and even heart failure. Among the primary mechanisms of chemotherapy‐induced cardiac injury are cardiac ferroptosis and PANoptosis triggered by oxidative stress. The novel nanozyme with antioxidant properties, developed for targeted cardiac delivery through the conjugation of TA and ANP, was shown to effectively alleviate DIC. Both BMzyme and ATBMzyme demonstrated significant efficacy in this regard. Further research confirmed that the nanozyme mitigated adriamycin‐induced cytotoxicity by counteracting ferroptosis and PANoptosis.

Intracellular iron accumulation drives ROS production and activates lipoxygenases, which damage both cell and mitochondrial membranes, leading to ferroptosis. Mitochondria‐targeted antioxidants have proven effective in mitigating DIC, thus highlighting that oxidative damage to mitochondria is a key mechanism in ferroptosis‐related cardiac injury.^[^ ^30^ ^]^ BMzyme and ATBMzyme, with their antioxidant activity, were initially hypothesized to have a protective role in DOX‐induced ferroptosis. Subsequently, both enzymes were shown to reduce ROS generation in cardiomyocytes, both in vitro and in vivo. They also decreased iron accumulation and lipid peroxidation in these cells, effectively restoring mitochondrial function compromised by DOX. Furthermore, PANoptosis, a cell death pathway associated with mitochondrial dysfunction,^[^ ^33^ ^]^ was identified. The nanozymes were able to lower the expression of ZBP1, a crucial component of the PANoptosome, thereby reducing DOX‐induced cardiomyopathy.^[^ ^34^, ^35^ ^]^ The nanozymes demonstrated significant protection not only against DIC but also against doxorubicin‐induced liver and kidney injuries. Importantly, this protection was achieved without compromising the tumor‐killing effect, presenting a novel approach to mitigating off‐target toxicity and side effects in tumor therapy.

## 3 Conclusion

This work demonstrates the effectiveness of a heart‐targeted and anchored bimetallic cluster nanozyme in alleviating chemotherapy‐induced cardiac ferroptosis and programmed necrosis. The novel nanozyme not only exhibits significant antioxidant and iron‐stress protective effects but also reduces inflammation and cell necrosis in cardiac cells or tissues, offering a promising new strategy for addressing chemotherapy‐related cardiac damage. Furthermore, the nanozyme has been shown to have minimal toxicity to cardiac tissue, good biocompatibility, and safety, establishing a strong foundation for its potential clinical application. Overall, this research provides both theoretical and experimental support for the development of advanced nanomaterials aimed at improving the quality of life and treatment outcomes for cancer patients. However, further research is needed to explore its molecular mechanisms and clinical application to fully validate and support its use in clinical settings.

## 4 Experimental Section

### Preparation and Characteristic of ATBMzyme—Synthesis of TBMzyme

Start by preparing a 25 mL methanol solution that contains 0.15 mmol of HAuCl_4_ and 0.10 mmol of RuCl_3_·H_2_O. Add 0.0612 g of N‐acetyl cysteine and 0.0153 g of tannic acid (TA) to the methanol solution. Next, add a cold NaBH_4_ aqueous solution to the mixture and stir at room temperature for 1 h. Then, continue stirring at 60 °C for 2 h, centrifuge to collect the precipitate, and freeze‐dry for later use.

### Preparation and Characteristic of ATBMzyme—Synthesis of ATBMzyme

Dissolve 10 mg of TBMzyme in 2 mL of aqueous solution, and add 5 mg of EDC and 4 mg of NHS. After stirring for 20 min, add 0.2 mg of ANP. Stir the mixture at room temperature overnight and collect the precipitate by centrifugation.

### Study Approval

This study was approved by the ethics committee of Central China Subcenter of National Center for Cardiovascular Diseases (Approval number FZX‐IACUC‐2024004). All animal procedures were conducted in accordance with the procedures reviewed and approved by the Care of Experimental Animals Committee. All efforts were made to minimize animal suffering.

### Statistics Analysis

Data were analyzed by the SPSS 16.0 software (SPSS, Inc., Chicago, IL, USA) and graphed using GraphPad Prism software (Version 8.0.2). Normality of distributions were tested by Shapiro‐Wilk test. Homogeneity of variance was determined by Levene's test. All continuous variables were presented as mean ± SD with a minimum of three independent experiments for in vitro experiments and six mice for in vivo experiments. For comparisons between two groups, two‐tailed Student's *t*‐tests were used. For comparisons among multiple groups, one‐way ANOVA followed by Tukey's post hoc test was used. *P* value < 0.05 was considered statistically significant. The specific statistical test for each experiment, data presentation and the meaning of the significance symbol for each experiment were indicated in the figure legends.

The detailed experimental processes are available in the Supporting Information.

## Conflict of Interest

The authors declare no conflict of interest.

## Supporting information



Supporting Information

## Data Availability

The data that support the findings of this study are available from the corresponding author upon reasonable request.

## References

[1] R. Jamaledin , C. K. Y. Yiu , E. N. Zare , L. N. Niu , R. Vecchione , G. Chen , Z. Gu , F. R. Tay , P. Makvandi , Adv. Mater. 2020, 32, 2002129. 10.1002/adma.202002129 32602146

[2] A. Lena , U. Wilkenshoff , S. Hadzibegovic , J. Porthun , L. Rösnick , A. K. Fröhlich , T. Zeller , M. Karakas , U. Keller , J. Ahn , L. Bullinger , H. Riess , S. D. Rosen , A. R. Lyon , T. F. Lüscher , M. Totzeck , T. Rassaf , D. Burkhoff , M. R. Mehra , J. J. Bax , J. Butler , F. Edelmann , W. Haverkamp , S. D. Anker , M. Packer , A. J. S. Coats , S. von Haehling , U. Landmesser , M. S. Anker , J. Am Coll. Cardiol. 2023, 81, 1569. 37076211 10.1016/j.jacc.2023.02.039

[3] M. S. Ewer , S. M. Ewer , Nat. Rev. Cardiol. 2015, 12, 547. 25962976 10.1038/nrcardio.2015.65

[4] M. Fan , H. Li , D. Shen , Z. Wang , H. Liu , D. Zhu , Z. Wang , L. Li , K. D. Popowski , C. Ou , K. Zhang , J. Zhang , K. Cheng , Z. Li , Adv. Sci. 2022, 9, e2203505. 10.1002/advs.202203505 PMC9661835 36058003

[5] K. T. Sawicki , V. Sala , L. Prever , E. Hirsch , H. Ardehali , A. Ghigo , Annu. Rev. Pharmacol. Toxicol. 2021, 61, 309. 33022184 10.1146/annurev-pharmtox-030620-104842

[6] K. T. Sawicki , A. De Jesus , H. Ardehali , Circ Res. 2023, 132, 379. 36730380 10.1161/CIRCRESAHA.122.321667 PMC9907000

[7] X. Fang , H. Ardehali , J. Min , F. Wang , Nat. Rev. Cardiol. 2023, 20, 7. 35788564 10.1038/s41569-022-00735-4 PMC9252571

[8] S. Sun , J. Shen , J. Jiang , F. Wang , J. Min , Signal Transduct Target Ther. 2023, 8, 372. 37735472 10.1038/s41392-023-01606-1 PMC10514338

[9] X. Yang , N. K. Kawasaki , J. Min , T. Matsui , F. Wang , J. Mol. Cell. Cardiol. 2022, 173, 141. 36273661 10.1016/j.yjmcc.2022.10.004 PMC11225968

[10] X. Fang , H. Wang , D. Han , E. Xie , X. Yang , J. Wei , S. Gu , F. Gao , N. Zhu , X. Yin , Q. Cheng , P. Zhang , W. Dai , J. Chen , F. Yang , H. T. Yang , A. Linkermann , W. Gu , J. Min , F. Wang , Proc. Natl. Acad. Sci. U S A 2019, 116, 2672. 30692261 10.1073/pnas.1821022116 PMC6377499

[11] T. Tadokoro , M. Ikeda , T. Ide , H. Deguchi , S. Ikeda , K. Okabe , A. Ishikita , S. Matsushima , T. Koumura , K. I. Yamada , H. Imai , H. Tsutsui , JCI Insight. 2020, 5, 132747. 32376803 10.1172/jci.insight.132747 PMC7253028

[12] Y. Wang , W. Gao , X. Shi , J. Ding , W. Liu , H. He , K. Wang , F. Shao , Nature 2017, 547, 99. 28459430 10.1038/nature22393

[13] M. E. Choi , D. R. Price , S. W. Ryter , A. M. K. Choi , JCI Insight 2019, 4, 128834. 31391333 10.1172/jci.insight.128834 PMC6693822

[14] X. Fang , Z. Cai , H. Wang , D. Han , Q. Cheng , P. Zhang , F. Gao , Y. Yu , Z. Song , Q. Wu , P. An , S. Huang , J. Pan , H. Z. Chen , J. Chen , A. Linkermann , J. Min , F. Wang , Circ. Res. 2020, 127, 486. 32349646 10.1161/CIRCRESAHA.120.316509

[15] Y. Xiong , X. Liu , C. P. Lee , B. H. Chua , Y. S. Ho , Free Radic. Biol. Med. 2006, 41, 46. 16781452 10.1016/j.freeradbiomed.2006.02.024

[16] H. C. Yen , T. D. Oberley , S. Vichitbandha , Y. S. Ho , D. K. St Clair , J. Clin. Invest. 1996, 98, 1253. 8787689 10.1172/JCI118909 PMC507548

[17] Y. Zhang , W. Yu , M. Chen , B. Zhang , L. Zhang , P. Li , Nanoscale 2023, 15, 12137. 37377098 10.1039/d3nr01722b

[18] J. Wu , X. Wang , Q. Wang , Z. Lou , S. Li , Y. Zhu , L. Qin , H. Wei , Chem. Soc. Rev. 2019, 48, 1004. 30534770 10.1039/c8cs00457a

[19] H. Fan , R. Zhang , K. Fan , L. Gao , X. Yan , ACS Nano 2024, 18, 2533. 38215476 10.1021/acsnano.3c07680

[20] J. Ge , L. Yang , Z. Li , Y. Wan , D. Mao , R. Deng , Q. Zhou , Y. Yang , W. Tan , J. Hazard. Mater 2022, 436, 129199. 35643002 10.1016/j.jhazmat.2022.129199

[21] W. Qiao , J. Chen , H. Zhou , C. Hu , S. Dalangood , H. Li , D. Yang , Y. Yang , J. Gui , Adv. Sci. 2024, 14, e2305979. 10.1002/advs.202305979 PMC11005736 38308189

[22] X. Wu , Y. Li , M. Wen , Y. Xie , K. Zeng , Y. N. Liu , W. Chen , Y. Zhao , Chem. Soc. Rev. 2024, 53, 2643. 38314836 10.1039/d3cs00673e

[23] Y. Zhang , W. Liu , X. Wang , Y. Liu , H. Wei , Small 2023, 19, e2204809. 36192166 10.1002/smll.202204809

[24] H. Li , J. Yan , D. Meng , R. Cai , X. Gao , Y. Ji , L. Wang , C. Chen , X. Wu , ACS Nano 2020, 14, 12854. 32955857 10.1021/acsnano.0c03629

[25] M. B. Kolli , N. Manne , R. Para , S. K. Nalabotu , G. Nandyala , T. Shokuhfar , K. He , A. Hamlekhan , J. Y. Ma , P. S. Wehner , L. Dornon , R. Arvapalli , K. M. Rice , E. R. Blough , Biomaterials 2014, 35, 9951. 25224369 10.1016/j.biomaterials.2014.08.037 PMC4758344

[26] L. Yang , H. Wang , X. Yang , Q. Wu , P. An , X. Jin , W. Liu , X. Huang , Y. Li , S. Yan , S. Shen , T. Liang , J. Min , F. Wang , Signal Transduct. Target Ther. 2020, 5, 138. 32732975 10.1038/s41392-020-00253-0 PMC7393508

[27] J. M. Madeira , D. L. Gibson , W. F. Kean , A. Klegeris , Inflammopharmacology 2012, 20, 297. 22965242 10.1007/s10787-012-0149-1

[28] X. Mu , J. Wang , Y. Li , F. Xu , W. Long , L. Ouyang , H. Liu , Y. Jing , J. Wang , H. Dai , Q. Liu , Y. Sun , C. Liu , X. D. Zhang , ACS Nano 2019, 13, 1870. 30753061 10.1021/acsnano.8b08045

[29] X. Liu , B. Chen , J. Chen , X. Wang , X. Dai , Y. Li , H. Zhou , L. M. Wu , Z. Liu , Y. Yang , Adv. Mater. 2023, 36, 2308477. 10.1002/adma.202308477 37985164

[30] J. Pan , W. Xiong , A. Zhang , H. Zhang , H. Lin , L. Gao , J. Ke , S. Huang , J. Zhang , J. Gu , A. C. Y. Chang , C. Wang , Adv. Sci. 2023, 10, e2206007. 10.1002/advs.202206007 PMC10214246 36967569

[31] A. R. Lyon , N. Yousaf , N. M. L. Battisti , J. Moslehi , J. Larkin , Lancet Oncol. 2018, 19, e447. 30191849 10.1016/S1470-2045(18)30457-1

[32] G. Curigliano , D. Cardinale , S. Dent , C. Criscitiello , O. Aseyev , D. Lenihan , C. M. Cipolla , CA Cancer J. Clin 2016, 66, 309. 26919165 10.3322/caac.21341

[33] Y. Bi , H. Xu , X. Wang , H. Zhu , J. Ge , J. Ren , Y. Zhang , Cell Death Disease 2022, 13, 1020. 36470869 10.1038/s41419-022-05460-x PMC9723119

[34] M. Zheng , T. D. Kanneganti , Immunol. Rev. 2020, 297, 26. 32729116 10.1111/imr.12909 PMC7811275

[35] Y. Lei , J. J. VanPortfliet , Y. F. Chen , J. D. Bryant , Y. Li , D. Fails , S. Torres‐Odio , K. B. Ragan , J. Deng , A. Mohan , B. Wang , O. N. Brahms , S. D. Yates , M. Spencer , C. W. Tong , M. W. Bosenberg , L. C. West , G. S. Shadel , T. E. Shutt , J. W. Upton , P. Li , A. P. West , Cell 2023, 186, 3013. 37352855 10.1016/j.cell.2023.05.039 PMC10330843

[36] Y. Bi , H. Xu , X. Wang , H. Zhu , J. Ge , J. Ren , Y. Zhang , Cell Death & Disease 2020, 13, 10.1038/s41419-022-05460-x. PMC9723119 36470869

