# Supplementary material for: A Cardiac‐Targeting and Anchoring Bimetallic Cluster Nanozyme Alleviates Chemotherapy‐Induced Cardiac Ferroptosis and PANoptosis
Source: Adv Sci (Weinh). 2024 Oct 28;12(1):2405597. doi: 10.1002/advs.202405597 (PMC11714205; doi:10.1002/advs.202405597)
Supplement: Supplementary file 1 — Supporting Information [file ADVS-12-2405597-s001.docx]

**Supporting Information**

**A Cardiac-Targeting and Anchoring Bimetallic Cluster Nanozyme Alleviates Chemotherapy-Induced Cardiac Ferroptosis and PANoptosis**

Junyue Xing^#^, Xiaohan Ma^#^, Yanan Yu^#^, Yangfan Xiao, Lu Chen, Yingying Wang, Keyu Liu, Zhiping Guo, Hao Tang*, Kelong Fan*, Wei Jiang*

Dr. J. Xing, Dr. X. Ma, M.S. Y. Yu, M.M. Y. Xiao, M.M. Y. Wang, Prof. Z. Guo, Prof. H. Tang, and Prof. W. Jiang

National Health Commission Key Laboratory of Cardiovascular Regenerative Medicine, Central China Subcenter of National Center for Cardiovascular Diseases, Henan Cardiovascular Disease Center, Fuwai Central-China Cardiovascular Hospital, Central China Fuwai Hospital of Zhengzhou University, Zhengzhou 450046, China.

Henan Key Laboratory of Chronic Disease Management, Central China Fuwai Hospital of Zhengzhou University, Zhengzhou, Henan, 451464, China.

Zhengzhou Key Laboratory of Cardiovascular Aging, Central China Fuwai Hospital of Zhengzhou University, Zhengzhou, Henan, 451464, China.

Email: [tangpku_zzuhao@zzu.edu.cn](mailto:tangpku_zzuhao@zzu.edu.cn) (H. T); [weijiang@zzu.edu.cn](mailto:weijiang@zzu.edu.cn) (W. J).

M.M. L. Chen

Department of Cardiovascular Diseases the First Clinical Medical College, Shanxi Medical University, Taiyuan, Shanxi, 030001, China.

B.S. K. Liu

School of Clinical Medicine, Shandong Second Medical University, Weifang, Shandong, 261053, China.

Prof. K. Fan

CAS Engineering Laboratory for Nanozyme, Key Laboratory of Biomacromolecules (CAS), CAS Center for Excellence in Biomacromolecules, Institute of Biophysics, Chinese Academy of Sciences, Beijing 100101, China.

Nanozyme Laboratory in Zhongyuan, Henan Academy of Innovations in Medical Science, Zhengzhou, Henan, 451163, China.

Email: [fankelong@ibp.ac.cn](mailto:fankelong@ibp.ac.cn) (K. F.).

**Experimental Section**

**Materials.**

1-(3-Dimethylaminopropyl)-3-ethylcarbodiimide hydrochloride (EDC), N-Hydroxysuccinimide;1-hydroxypyrrolidine-2,5-dione (NHS), RuCl_3_·H_2_O, chloroauric acid (HAuCl_4_), N-acetyl cysteine, DPPH, ABTS, and H_2_O_2_ was purchased from Sigma-Aldrich. ANP was puechased from Sangon Biotech, Shang hai, and the protein sequence is SLRRSSCFGGRMDRIGAQSGLGCNSFRY. The human embryonic kidney 293 (HEK293) cell line was purchased from iCell Bioscience Inc, Shanghai. The HEK293 cells were cultured in Dulbecco’s modified Eagle’s medium (DMEM) supplemented with 10% fetal bovine serum (FBS), 100 μg mL^−1^ streptomycin, and 100 U mL^−1^ penicillin at 37°C in an incubator supplied with a humidified atmosphere of 5% CO_2_.

**Characterizations.**

The morphology of ATBMzyme was observed by TEM (HT7700, Hitachi, Japan). The zeta potential of nanozymes were determined by Nanosizer Pro (Malvern Instruments, Britain). ESR spectroscopy was carried out by a ESR spectrometer (A300-10/12, Bruker, Germany), 2,2-dimethyl-1-oxido-3,4-dihydropyrrol-1-ium (DMPO) and Triacetonamine (TEMP) were used as superoxide radical and singlet oxygen catching agent, respectively. XPS was carried out by XPS (Thermo Scientific K-Alpha, USA). XRD spectroscopy was carried out by a XRD spectrometer (Bruker D8 Advance, Bruker, Germany). The ^1^H nuclear magnetic resonance (NMR) spectra were obtained on a Bruker Avance III NMR spectrometer (400 MHz) with D_2_O as the solvent, in which chemical shifts (in ppm) were recorded using tetramethylsilane as an internal standard.

**CAT-like activity assay of BMzyme, TBMzyme, and ATBMzyme**: To assess the oxygen-production capacity of BMzyme, TBMzyme, and ATBMzyme, samples were dispersed into10 mL aqueous solution containing 0.1 mM H_2_O_2_, and adissolved oxygen meter was used to test the O_2_ generation.

**SOD-like activity of BMzyme, TBMzyme, and ATBMzyme**: The SOD-like activity of samples was determined using xanthine oxidase method. Briefly 30 μL of prepared nanozyme solutions (0.0015625 ⁓ 0.1 mg/mL) were transferred into a 96-well plate. xanthine, pH = 7.4 phosphate buffer, cytochrome C, xanthine oxidase working solution were added in sequence. The resulting solutions were immediately measured the absorbance of each well at 450 nm for 2 minutes before and after using a microplate reader, and the inhibition percentage was calculated.

**ABTS radical-scavenging activity assay of the BMzyme, TBMzyme, and ATBMzyme:** ABTS solution (7 mM) was first incubated with potassium persulfate (2.45 mM) overnight for activating ABTS radicals. BMzyme, TBMzyme, and ATBMzyme with a final concentration of (1.5625 ⁓ 100 μg/mL were added into ABTS radical solutions. The time-course absorption of ABTS radicals was measured at 734 nm for 6 min. ABTS scavenging percentage = (A_0_-A_sample_)/A_0_ × 100%. A_0_ is the absorbance of ABTS at 520 nm when no sample is added; A sample is the absorbance of the ABTS added to the sample at 520 nm.

**DPPH radical scavenging activity assay of the BMzyme, TBMzyme, and ATBMzyme:** Determine the DPPH radical scavenging rate according to the protocol established in the literature. In short, after dissolving DPPH in anhydrous ethanol, store it in dark, and then mix the fresh DPPH solution with different concentrations of BMzyme, TBMzyme, and ATBMzyme (1.5625 ⁓ 100 μg/mL). Incubate at 37° C in darkness for 30 minutes, then record the absorbance at 520 nm using a Microporous Plate Reader and calculate the cleared DPPH. The calculation formula is the expression of free radical scavenging ability.

**Density Functional Theory (DFT) calculations:** The spin-polarized density functional theory (DFT) calculations have been conducted on Vienna ab-initio simulation package (VASP)[1, 2] to study the catalytic properties of prepared catalysts. The Projector augmented wave method[2] with a cutoff energy of 400 eV accompanied by Perdew-Burke-Ernzerhof functional[3] has been used in the DFT calculations. DFT-D3 method[4] was used to correct the influence of van der Waals interactions. An AuRu alloy particle has been built by 17 of Ru-atoms and 26 of Au-atoms and put in a cubic box with the length of 25 Å. All models have been fully relaxed with the energy convergence criterion of 10 - 5 eV and the force convergence criterion of 0.02 eV/Å, respectively. The Brillouin zone integration was performed with the K-point mesh of the Γ point. The adsorption energy (Eads) has been calculated using formula 1,

E_ads=E_total-E_substrate-E_adsorbate (1)

The Etotal, Esubstrate and Eadsorbate represent the energy of adsorption structure, substrate and adsorbate, respectively. The free energies have been calculated using the following formula 2,

G= E_DFT+ZPE-TS (2)

The G, EDFT, ZPE and TS represent the free energy, energy from DFT calculations, zero point energy and entropic contributions, respectively.

**Cell culture and treatment:** AC16 cardiomyocytes were cultured in Hyclone high-glucose DMEM supplemented with 10% FBS and 1% antibiotics at 37°C in 5% CO_2_. When these cells reached 40 - 60% confluence, they were treated with TBMzyme for 20 hours, and then changed culture medium and incubated with doxorubicin (DOX) for 24 h.

**Cell viability measurement:** AC16 cell viability was measured by propidium iodide (PI) staining (Biosharp, China). AC16 was digested with trypsin and washed twice with PBS, followed by PI staining for 15 minutes. The proportion of PI-positive cells was analyzed by BD [FACSCantoⅡ](http://www.baidu.com/link?url=mm8ZqEffDz876JHT8a6m4ZHbf-SV-If9OBHdwEtPaiGIoPlATcL0Clcli86Pz15nVNHRHhqSJqbVZ7e1A__KkUxnX_kvRNmGftG52UpAV-JR7Ty8w5sDCACFcRDCBnFGe1-p8NaGDEWPu8WnA2F2mnMT4nAsqnwC20SD4ZCgJnQKqE1YmCyeMQfLiATnIaqz_9Vo1Rx5eiD2td_t_kcgiOi9y9JZIqIQeBn-ubhOCF0F_3_Et4894DeioSc4MkIGR8gppWOjNbAZ1l80EdTxf0ajYvpJhYaKakjE8hk-BDk4smJkcLvvnzEtTBxqUiBuQg0R6mU7xMshcFnAHGk0UE7EfwThqFj5OBoZMiCv9g4k7IjvcqXvrqRFSybYbawRMjxMbrclwwY1QRkoZENk3Y0YzZb358KXsy-jeLjx3F_) flow cytometry (BD Biosciences, USA).

**Lysosome escape.** The lysosome escape behaviors of TBMzyme were studied in AC16 cells. The AC16 cells were seeded in a 12-well plate and incubated at 37℃ for 12 h. Thereafter, the Cy5.5-labeled TBMzyme were added and incubated at 37℃ for predetermined periods of time. The lysosome was added to the wells according to the manufacture’s instructions (75 nM LysoTracker Green; 30 min at 37°C, Beyotime Co. Ltd., Shanghai, china).Then,the cells were washed in PBS and stained with DAPI, and coverslips were mounted on clean glass slides.Last, the slides were imaged using a confocal microscope (Leica, Germany). Different fluorescent pictures were analyzed by software LAS X.

**Mitochondrial membrane potential analysis:** JC-1 staining kit (Beyotime, China) was used to detect Mitochondrial membrane potential. Briefly, treated AC16 cells were stained with JC‐1 staining solution and then incubated in a 37°C cell incubator with 5% CO_2_ for 20 min. Afterwards, the cells were washed twice with JC‐1 staining buffer and DAPI was used for nuclei staining. Fluorescence images were captured by Nikon Confocal microscopy (Nikon, Japan).

**Intracellular ROS level analysis:** The level of reactive oxygen species (ROS) was analyzed by a cellular ROS assay kit (Beyotime, China). Briefly, treated AC16 cells were stained with 10 μmol/L DCFH-DA probes for 20 min in a 37°C cell incubator，then the cells were washed three times with serum-free cell culture medium, after that, cells were digested with trypsin and resuspended with PBS for flow cytometry analysis using [FACSCantoⅡ](http://www.baidu.com/link?url=mm8ZqEffDz876JHT8a6m4ZHbf-SV-If9OBHdwEtPaiGIoPlATcL0Clcli86Pz15nVNHRHhqSJqbVZ7e1A__KkUxnX_kvRNmGftG52UpAV-JR7Ty8w5sDCACFcRDCBnFGe1-p8NaGDEWPu8WnA2F2mnMT4nAsqnwC20SD4ZCgJnQKqE1YmCyeMQfLiATnIaqz_9Vo1Rx5eiD2td_t_kcgiOi9y9JZIqIQeBn-ubhOCF0F_3_Et4894DeioSc4MkIGR8gppWOjNbAZ1l80EdTxf0ajYvpJhYaKakjE8hk-BDk4smJkcLvvnzEtTBxqUiBuQg0R6mU7xMshcFnAHGk0UE7EfwThqFj5OBoZMiCv9g4k7IjvcqXvrqRFSybYbawRMjxMbrclwwY1QRkoZENk3Y0YzZb358KXsy-jeLjx3F_) flow cytometry (BD Biosciences, USA). The ROS content in mouse heart was determined using the Mouse ROS ELISA Kit (YJ112563,mlBio) according to the manufacturer’s instructions.

**Glutathione (GSH) and Glutathione Disulfide (GSSG) level assays:** Following the manufacturer's instructions, the GSH and GSSG Assay kit (Beyotime, China) was used to measure intracellular GSH. After cell harvesting and PBS washing, 3 times cell pellet volume of Deproteinizing Reagent M solution was added. The samples were then put through two quick freeze-thaw cycles using liquid nitrogen and water heated to 37°C. The samples were centrifuged for 10 minutes at 10,000 g after being placed on ice for 5 minutes to determine the amount of total glutathione in the supernatant. Additionally, the aforementioned supernatant was pipetted with one-fifth the volume of the GSH removal working solution and one-twenty-fifth the volume of the auxiliary solution, respectively. The solutions were mixed using a vortex and incubated at 25°C for one hour to produce samples for GSSG determination. In a 96-well plate, 10 μL of the supernatant was added with 150 μL of the prepared total glutathione detection working solution and incubated at 25°C for 5 min. Then, 50 μL of NADPH solution (0.5 mg/mL) was added. After reacting for 25 min, the microplate reader measured the absorbance at a wavelength of 412 nm for each well. GSSG was conducted in the same manner. The following formula was used to further compute the GSH and GSSG levels: GSH = Total Glutathione-GSSG × 2.

**Intracellular MDA level assay:** The content of MDA was detected by the Lipid Peroxidation MDA Assay kit (Beyotime, China). Cells were collected and lysed with RIPA lysis buffer (Applygen, China), after centrifugation at 12,000 g for 15 min, the supernatant was collected and used for the enzyme assays, the MDA content was measured with a spectrophotometer at a wavelength of 532 nm.

**Intracellular Fe^2+^ level analysis:** Divalent iron levels of treated AC16 cells were determined by FerroOrange staining (DOJINDO, Japan), followed by DAPI staining. The fluorescence was photographed using Nikon fluorescent microscope (Nikon, Japan).

**Mito Fe^2+^ staining:** To assess mitochondrial Fe^2+^ content, cells were incubated with Mito-FerroGreen (M489, Dojindo) following the manufacturer's protocol. Specifically, the treated AC16 cells were washed three times with Hank's Balanced Salt Solution (HBSS) and subsequently incubated with 5 μM Mito-FerroGreen in HBSS in a 37°C cell incubator with 5% CO_2_ for 30 min. Nuclei were stained by DAPI. Following three washes with phosphate-buffered saline (PBS), fluorescence was observed using confocal microscope (Nikon, Japan). Fluorescence intensity was quantified using ImageJ software.

**MitoPeDPP staining:** Lipid peroxidation was measured using MitoPeDPP (M466, Dojindo ) according to the manufacturer’s instructions. In brief, the treated AC16 cells were washed three times with PBS and were stained with 0.1 μM MitoPeDPP for 20 min in a 37°C cell incubator.Then, the cells were washed three times with PBS and DAPI was used for nuclei staining. Fluorescence images were captured by Nikon Confocal microscopy (Nikon, Japan). ImageJ software was used to quantify fluorescence intensity.

**Western blot Analysis:** Western blot analysis was performed in accordance with standard procedures. Briefly, cells or tissue were lysed with RIPA (adding protease inhibitors), and centrifugated at 12 000 rpm for 15 min at 4°C. Then, protein lysates were transferred to a new 1.5 mL Eppendorf tube, and the protein concentrations were determined using the bicinchoninic acid (BCA) assay, protein samples were fractionated by sodium-dodecyl-sulfate polyacrylamide gel electrophoresis (SDS-PAGE) and transferred onto nitrocellulose membranes (Millipore). These Membranes were blocked in a blocking buffer (5% skimmed milk in TBS-T) for 1 hour and incubated with different primary antibodies: ZBP1 (proteintech, Cat#13285-1-AP, 1:1000), caspase-3 (HUABIO, Cat#ET1608-64, 1:1000), cleaved caspase-3 (CST, Cat#9664, 1:1000), MLKL (proteintech, Cat#66675-1-Ig, 1:2000), p-MLKL (abcam, Cat#ab196436, 1:1000), GSDMD (abcam, Cat#ab209845, 1:1000), cleaved GSDMD (HUABIO, Cat#HA721144, 1:1000), Vinculin (proteintech, Cat#66305-1-Ig, 1:8000), Tubulin (Beyotime, Cat#AF2827, 1:5000), SLC7A11 (proteintech, Cat#26864-1-AP, 1:5000), GCLM (proteintech, Cat#14241-1-AP, 1:5000), NQO1 (proteintech, Cat#11451-1-AP, 1:5000). Following incubation with the HRP-conjugated secondary antibody for 1 hour at room temperature. Protein bands were detected with an AI800 instrument (GE) using an ECL western blotting substrate (Millipore).

**Animal model and treatments:** Male Balb/c mice aged 6 to 8 weeks were obtained from the Institute of Laboratory Animal Science, Chinese Academy of Medical Sciences (Beijing, China) and fed adaptively for a week before the study's start. All mice were kept in a temperature-controlled environment with a 12-hour light/dark cycle at 22 ± 2°C, and were provided sterile food and drinking water..

To obtain tumor-bearing mouse models, mouse breast cancer cells 4T1 (8 × 10^5^/mouse) were subcutaneously implanted into the back of eight-week-old male mice. Tumor volume (in mm^3^) was measured with calipers and calculated as (length × width^2^) × 0.52 once every 3 days for 3 weeks. At the end of the experiment, the tumors were dissected and weighed for analysis.

To generate the chronic doxorubicin-induced cardiotoxicity (DIC) model, the aforementioned tumor-bearing mice were divided into four groups and received a weekly i.p. injection of DOX (5 mg/kg; MedChem Express, USA) for 4 weeks, these mice were given a weekly i.p. injection of TBMzyme or ATBMzyme (for the treatment of DIC) 6 hours before each DOX treatment.

***In vivo* bioluminescence imaging.** 6-8 weeks male Balb/c mice with tumor were intravenously (i.v.) injected with 10 mg/kg of TBMzyme, ABMzyme, ATBMzyme, and BMzyme through the lateral tail veins. After 24 h, bioluminescence image acquisition was performed using the IVIS Spectrum In Vivo Imaging System (PerkinElmer). All image postprocessing and analyses were performed using the Living Image 4.8.0 software (PerkinElmer). Finally, the heart, liver, lung, spleen, kidney and tumor of mice were harvested and fixed, sliced, stained with DAPI, and then observed under fluorescence microscope (Nikon).

**Echocardiography:** Transthoracic echocardiography was performed using a Visual Sonics Vevo 3000 system (FUJIFILM Visual Sonics, Japan). Mice were anesthetized and maintained under 1 ⁓ 2% isoflurane and 2 L min^−1^ 20% oxygen during the procedure. LVEF, LVFS were analyzed using Vevo Analysis software (FUJIFILM Visual Sonics, Japan).

**Serum measurement:** On the endpoint, we sacrificed mice of each groups, approximately 0.5 mL of blood was collected from each mouse via orbital bleeding. For serum biochemical analysis, the blood samples were centrifuged at 3000 rpm for 10 minutes. Then, the serum was collected from the supernatant, and the serum LDH, cTnT, CK-MB, NT-proBNP, ALT, AST, UREA and CREA levels were evaluated.

**Transmission electron microscopy:** Samples of myocardium (1 mm × 1 mm × 3 mm) were quickly removed from the left ventricle and immediately fixed in 3% phosphate glutaraldehyde. Then, the samples were post-fixed, embedded, cut, and mounted. The samples were observed under a transmission electron microscope (TEM) and images were taken.

**Histological examination:** For the histological examination of the heart, liver, kidney, lung, spleen, and tumor, the mice were sacrificed, and these tissues were paraffin-embedded for hematoxylin and eosin (H&E) staining, terminal deoxynucleotidyl transferase-mediated dUTP-biotin nick-end labeling (TUNEL), and 4-HNE immunohistochemistry (IHC) detection according to the protocols of the kit supplier.

**Supplementary Figures**


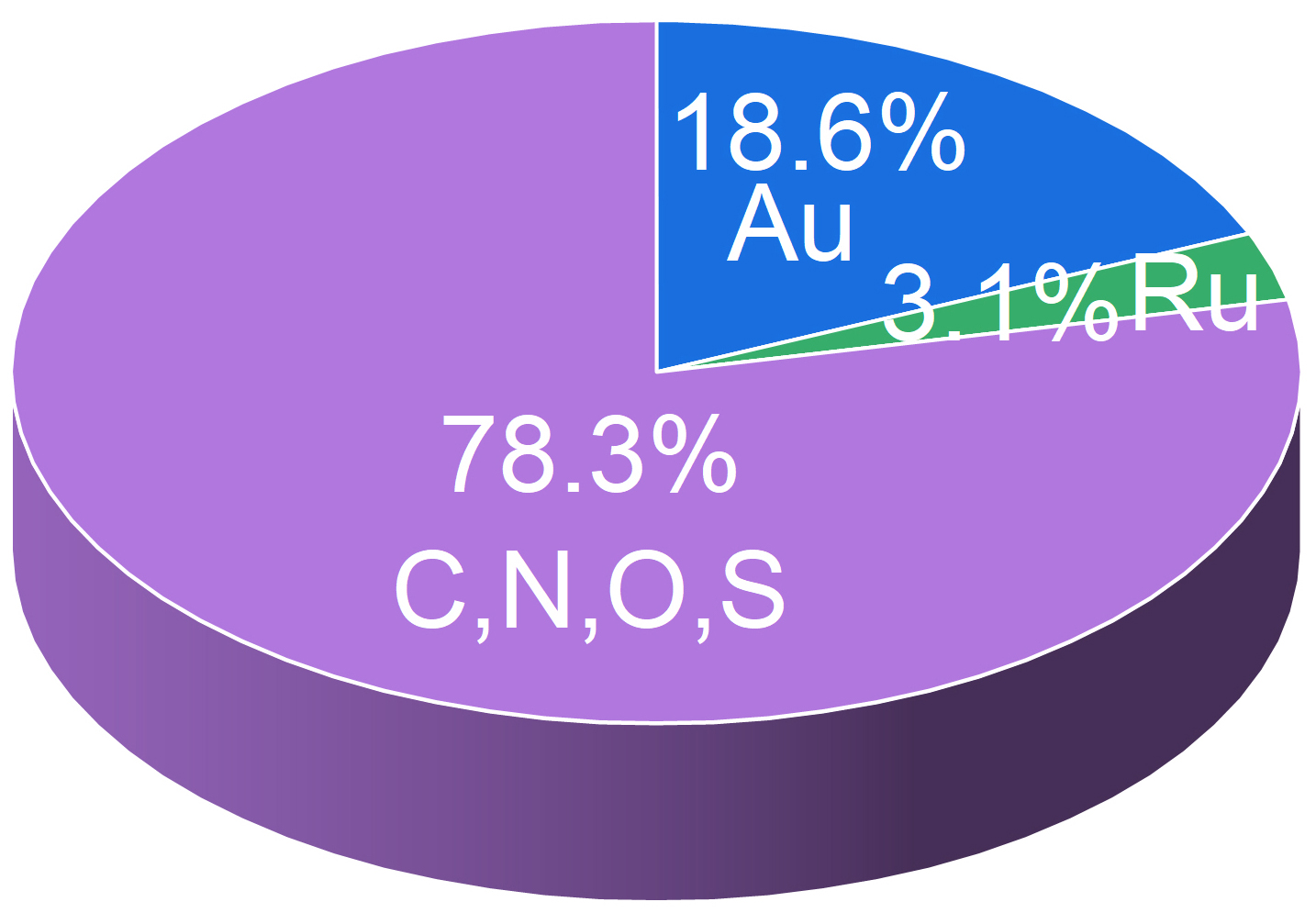


**Figure S1.** The proportion of elements in ATBMzyme detected by ICP-MS.

**
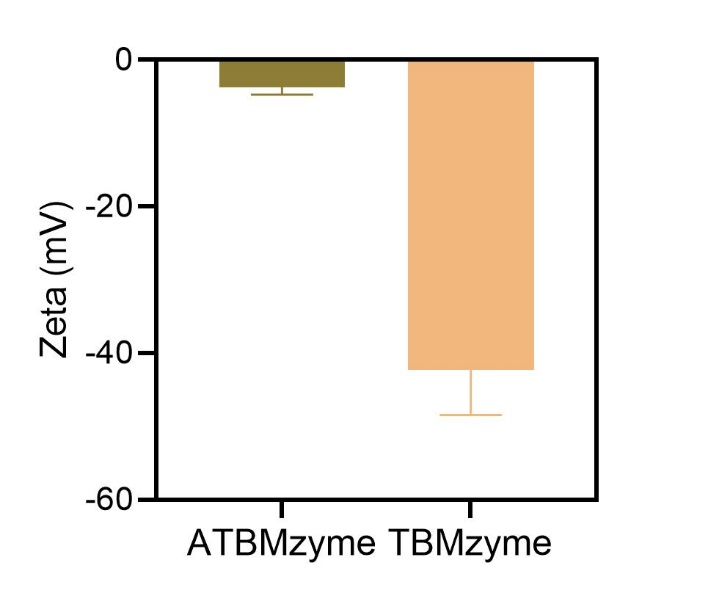
**

**Figure S2.** Zeta potential analyses of ATBMzyme and TBMzyme.


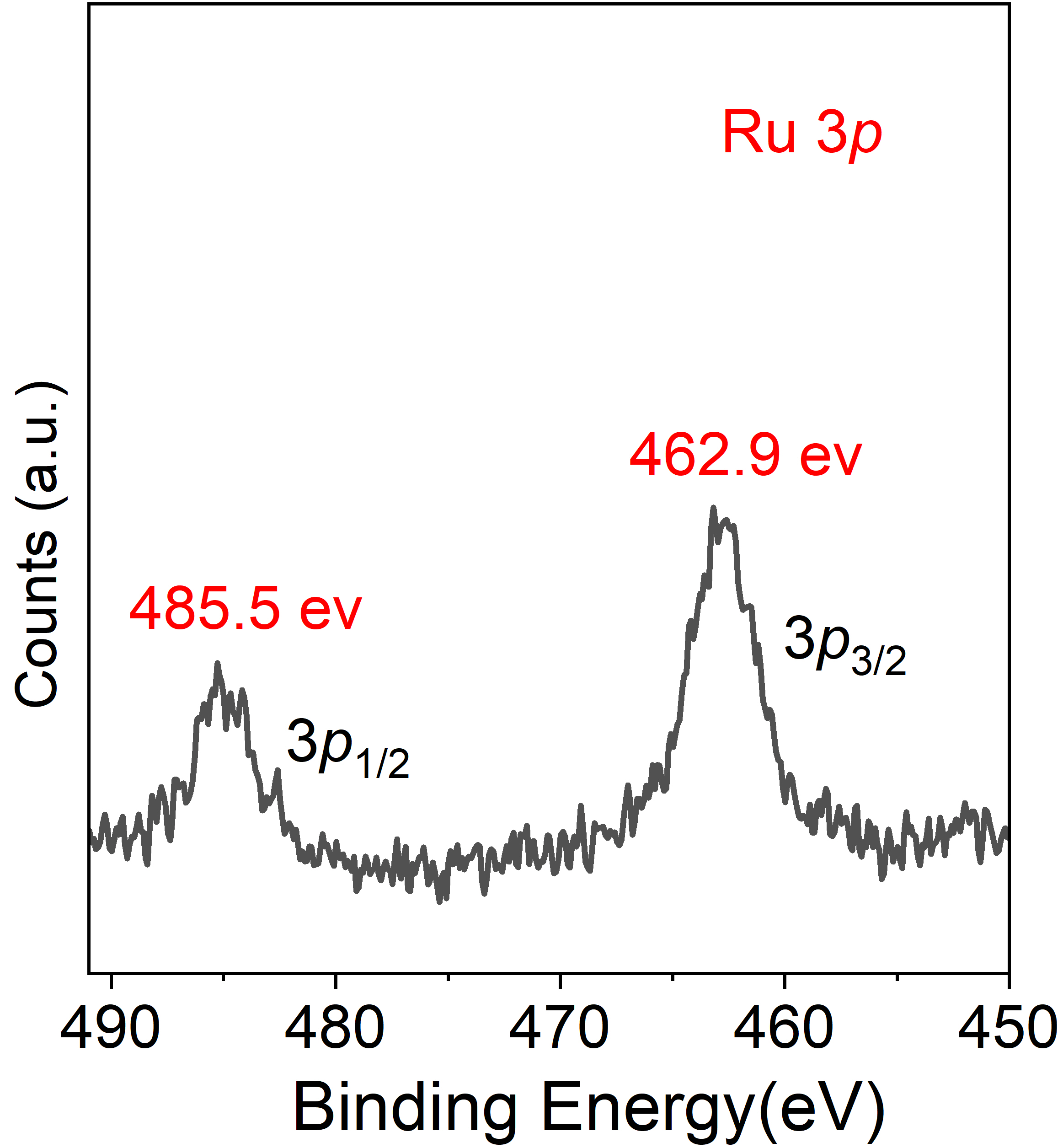


**Figure S3.** XPS spectra of Ru 3*p* in ATBMzyme


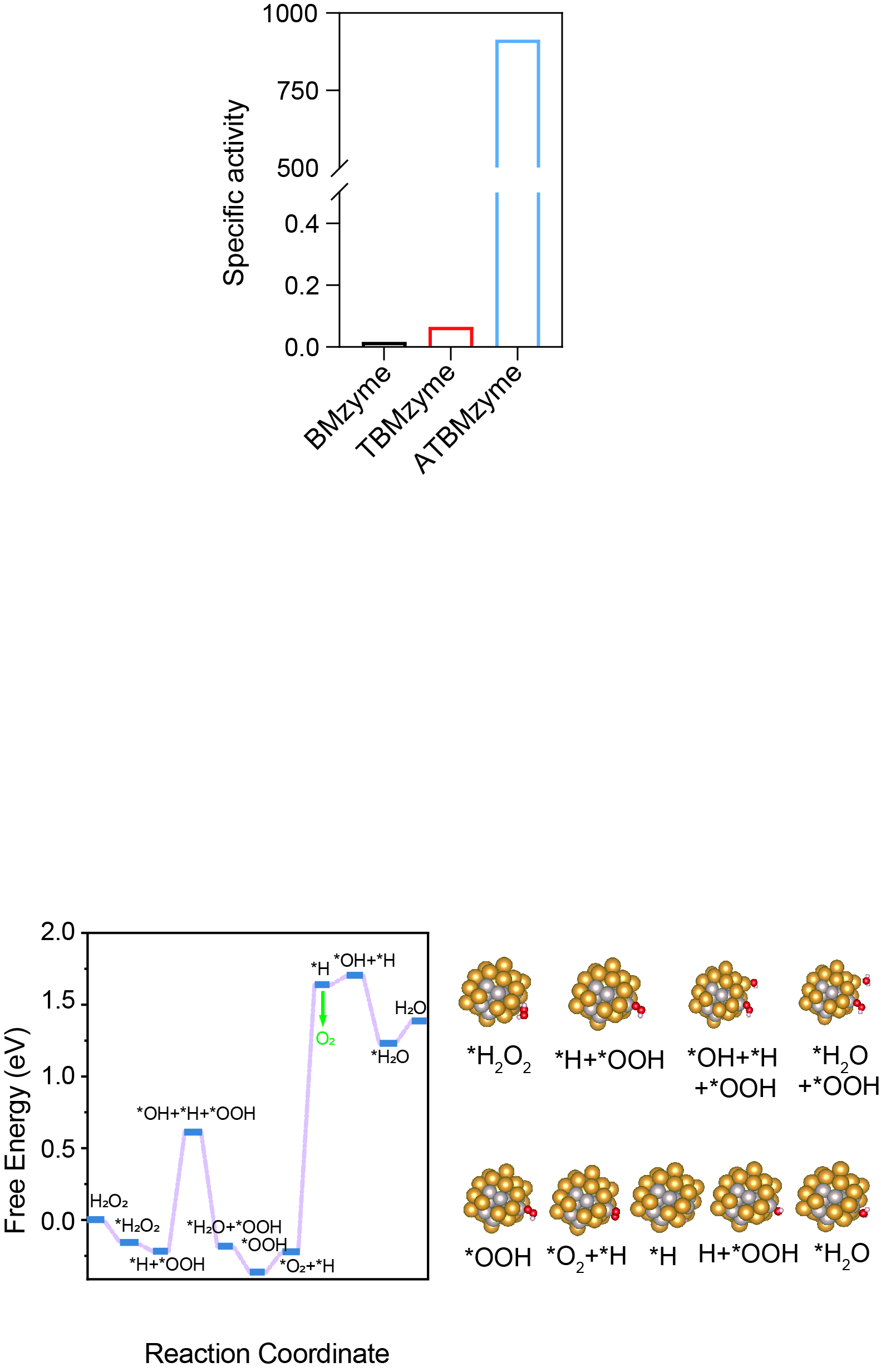


**Figure S4.** The SOD-like specific activity of BMzyme, TBMzyme, ATBMzyme for Figure 3B.


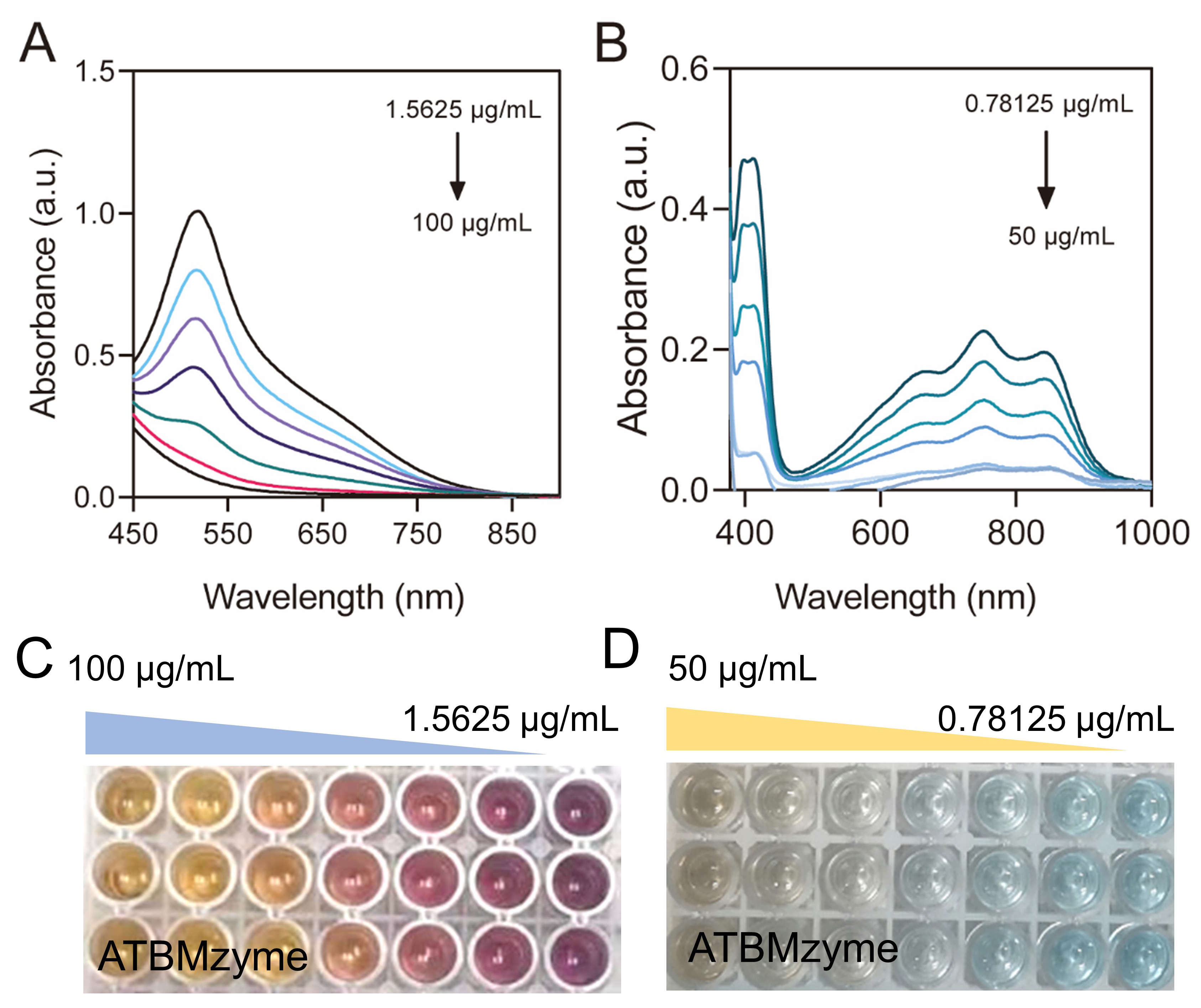


**Figure S5.** **Nitrogen free radical scavenging.** UV-vis spectra of DPPH• (A) and ABTS^•+^ (B) recorded after the addition of different concentration of ATBMzyme. Representative optical photograph of DPPH• (C) and ABTS^•+^ (D) after the addition of different concentration of ATBMzyme.


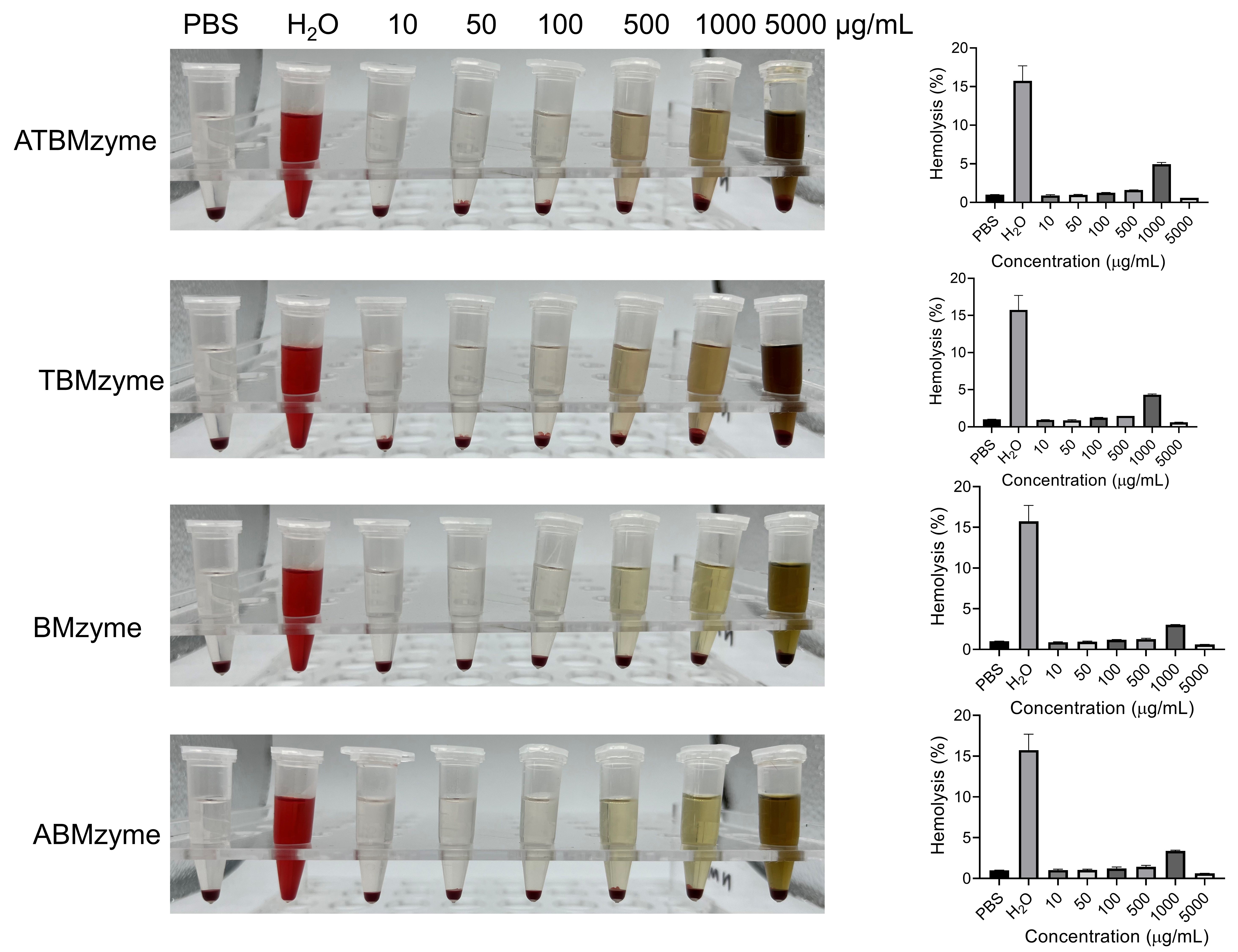


**Figure S6.** Visual observation of hemolysis (A) and hemolytic activity (B) of ATBMzyme, TBMzyme, BMzyme, and ABMzyme using DI water as control. Data were expressed as mean value ± SD of three experiments.


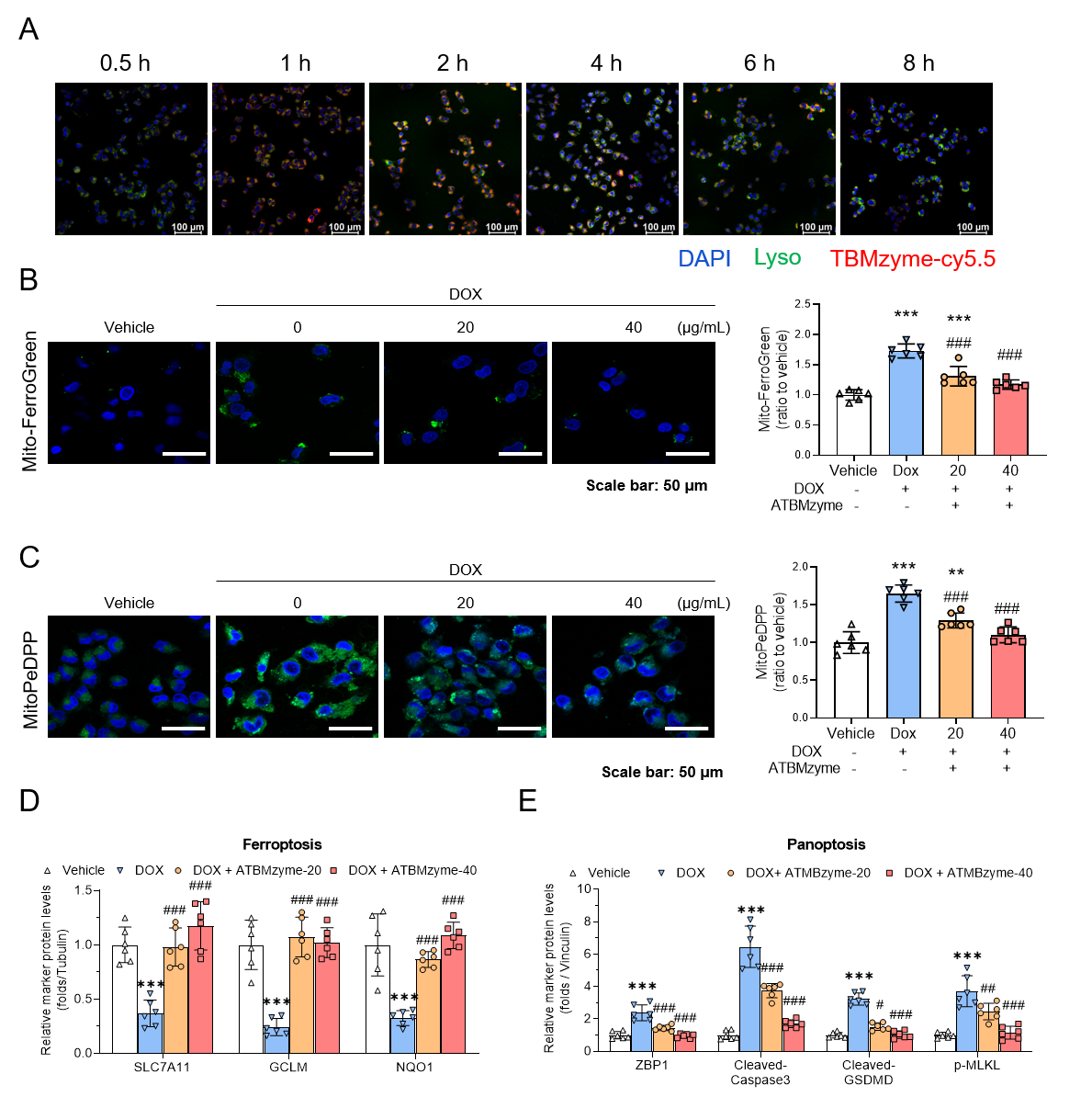


**Figure S7.** ATBMzyme mitigating ferroptosis and PANoptosis in myocardial cells. (A) Colocalization observation by CLSM of the cy5.5-labeled TBMzyme (red) and LysoTracker (green) in AC16 cells. Scale bars = 100 μm. (B) Representative micrographs (left) and quantitative analysis (right) of mitochondrial iron level in AC16 cells with different treatment (scale bars, 50 μm; n = 6). (C) Representative micrographs (left) and quantitative analysis (right) of mitochondrial lipid peroxidation level in AC16 cells with different treat (scale bars, 50 μm; n = 6). (D) Quantitative analysis of ferroptosis–related proteins in AC16 (n = 6). (E) Quantitative analysis of PANoptosis-related proteins in AC16 (n = 6). The data are expressed as mean ± SD and analyzed using one-way ANOVA followed by Tukey’s post hoc test, ***p* < 0.01 versus Vehicle group; ****p* < 0.001 versus Vehicle group; #*p* < 0.05, ##*p* < 0.01, and ###*p* < 0.001 versus DOX group.

**
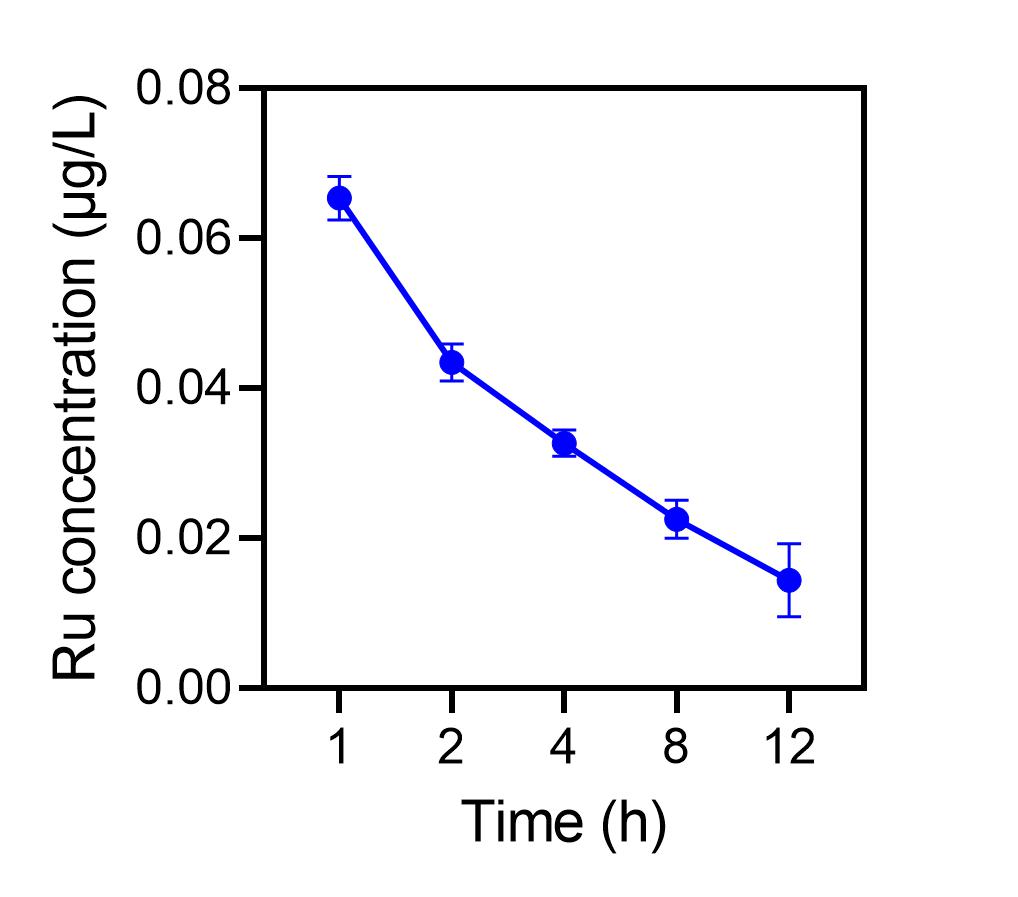
**

**Figure S8.** The pharmacokinetics of ATBMzyme, n=3.


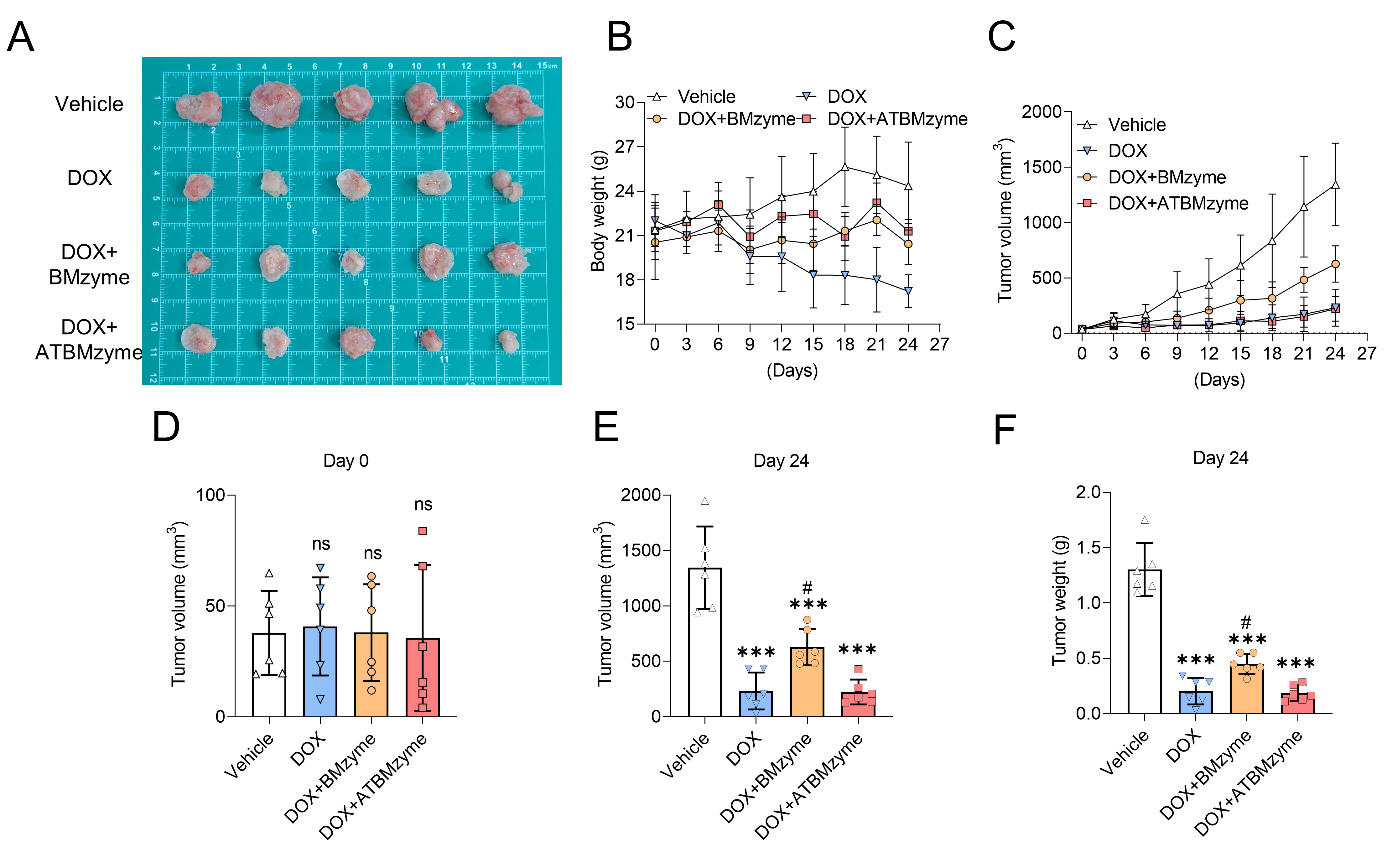


**Figure S9.** ATBMzyme did not dampen the anti-tumor effect of Doxorubicin, and ATBMzyme was specifically enriched in the heart. (A) Representative images of tumors procured at the end time point. (B) Body weight of mice (n = 6). (C) Tumor volume was measured every 3days, and were shown using a time-course line graph. (D) Quantitative analysis of tumor volume in day 0 (n = 6). (E) Quantitative analysis of tumor volume in day 24 (n = 6). (F) Quantitative analysis of tumor weight in day 24 after the mice was sacrificed (n = 6). The data are expressed as mean ± SD, multiple groups data analyzed using one-way ANOVA followed by Tukey’s post hoc test, ****p* < 0.001 versus Vehicle group; #*p* < 0.05 versus DOX group.


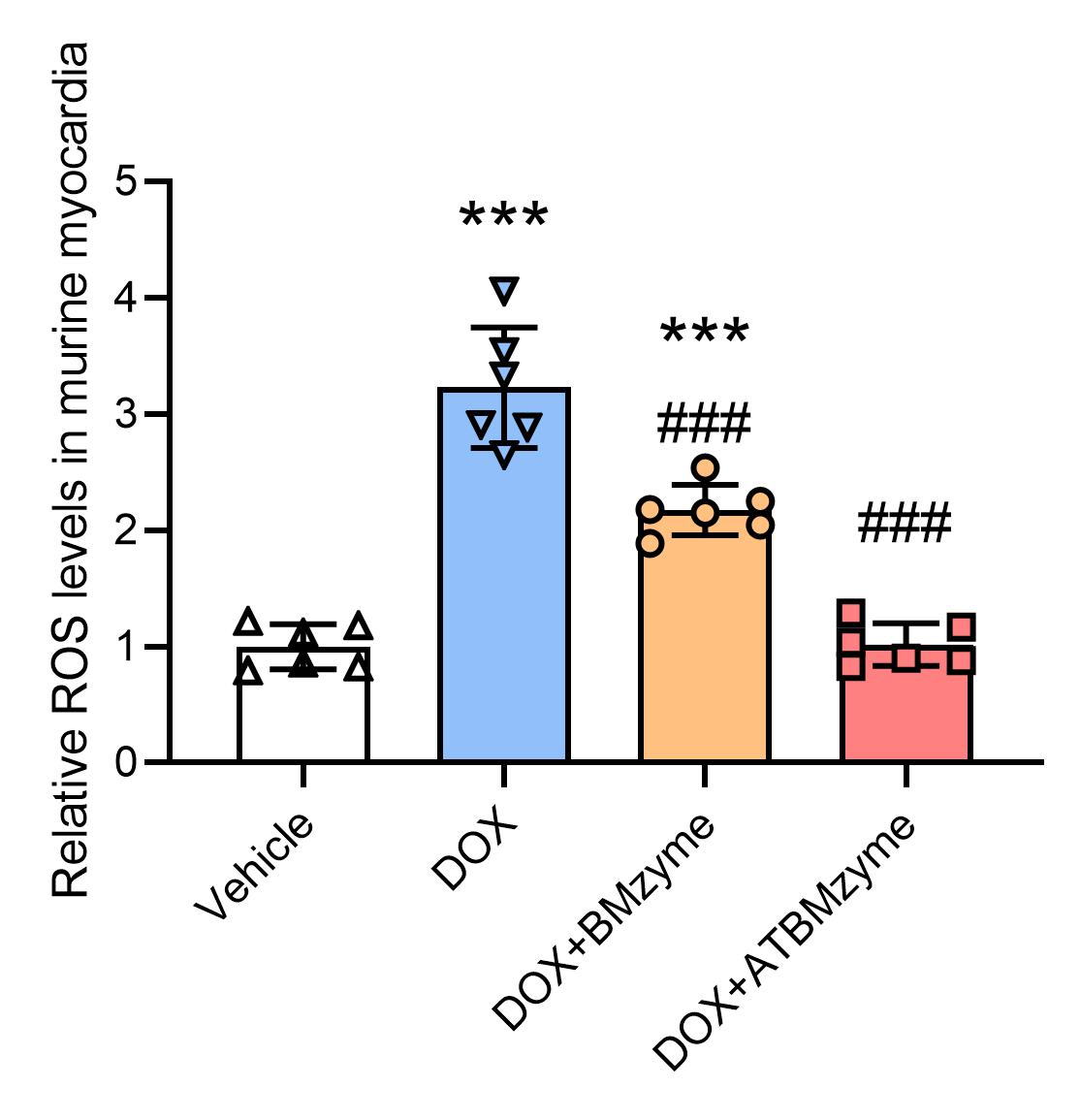


**Figure S10.** Total ROS levels in murine myocardia. Data were expressed as mean ± SD and analyzed using one-way ANOVA followed by Tukey’s post hoc test, ****p* < 0.001 versus Vehicle group; ###*p* < 0.001 versus DOX group.


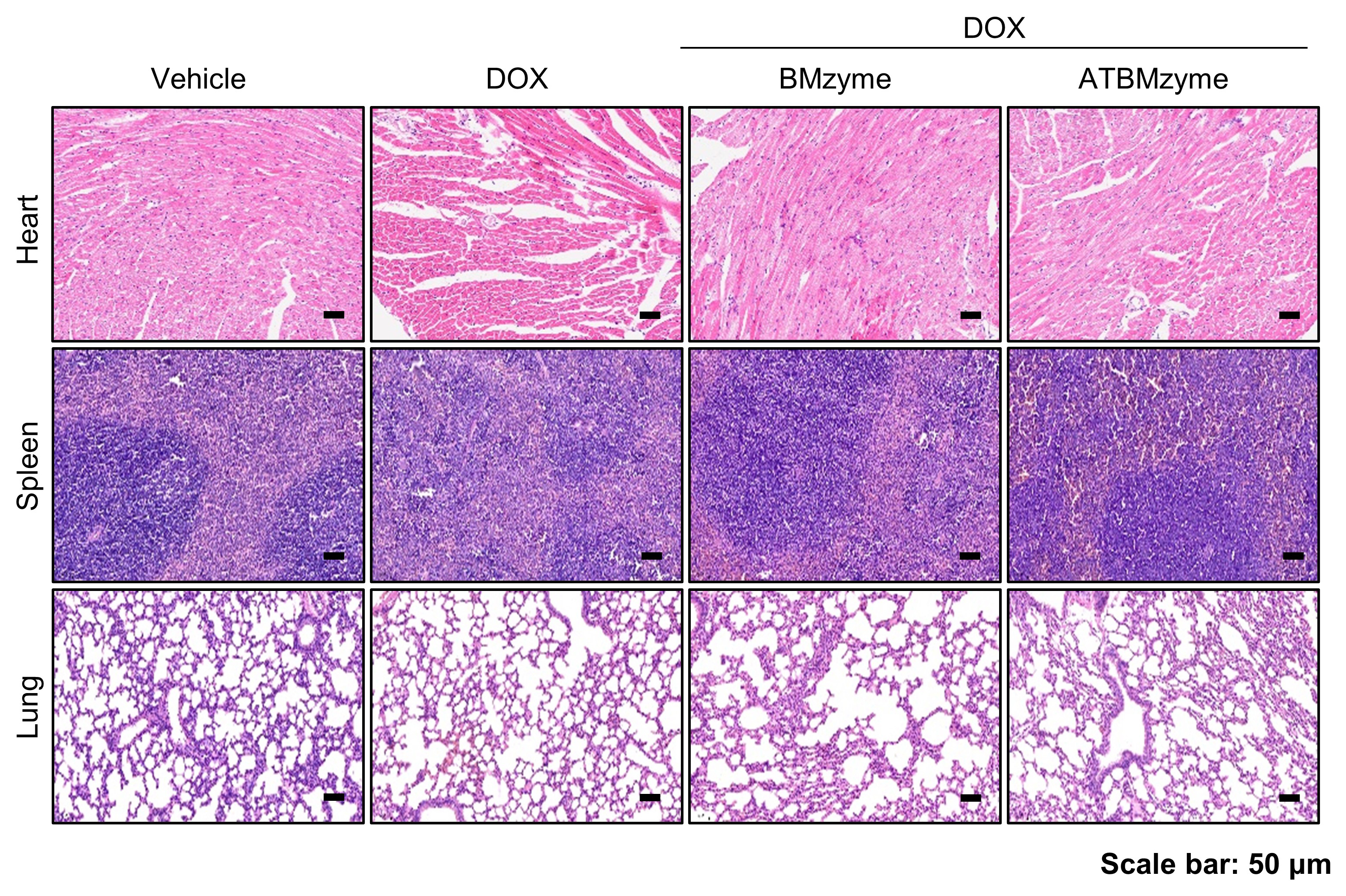


**Figure S11.** H&E staining was performed on lung, spleen, and heart tissues following treatment by Vehicle, DOX, DOX + BMzyme, and DOX + ATBMzyme (scale bar, 50 µm; n = 6).

**Reference**

1. Kresse, G., & Furthmüller, J. (1996). Efficient iterative schemes for ab initio total-energy calculations using a plane-wave basis set. Physical review B, 54(16), 11169.

2. Kresse, G., & Joubert, D. (1999). From ultrasoft pseudopotentials to the projector augmented-wave method. Physical review b, 59(3), 1758.

3. Perdew, J. P., Burke, K., & Ernzerhof, M. (1996). Generalized gradient approximation made simple. Physical review letters, 77(18), 3865.

4. Grimme, S., Antony, J., Ehrlich, S., & Krieg, H. (2010). A consistent and accurate ab initio parametrization of density functional dispersion correction (DFT-D) for the 94 elements H-Pu. The Journal of chemical physics, 132(15), 154104.
